# Oblique Quasi-Static Indentation and Residual Compressive Behavior of Hybrid Woven Composite Laminates

**DOI:** 10.3390/ma19163399

**Published:** 2026-08-10

**Authors:** Obinna Emmanuel Ebubechukwu, Guohui Chang, Qing Miao, Hiranya Uthpali Herath, Jiaxi Zhang, Deng’an Cai

**Affiliations:** 1 State Key Laboratory of Mechanics and Control for Mechanical Structures, College of Aerospace Engineering, Nanjing University of Aeronautics and Astronautics, Nanjing 210016, China; obinnaebube@nuaa.edu.cn (O.E.E.);; 2 AVIC Chengdu Aircraft Design & Research Institute, Chengdu 610091, China; 3 National Key Laboratory of Digital and Agile Aircraft Design, Chengdu 610091, China

**Keywords:** hybrid woven composites, stacking sequences, finite element, quasi-static indentation, compression after indentation, failure mechanism

## Abstract

The influence of oblique quasi-static indentation (QSI) angles on damage evolution and the residual compressive behavior of carbon–glass hybrid woven composite laminates is investigated. Hemispherical indentation tests were performed at 15° and 30° oblique angles, and three QSI specimens and two CAI (compression after Indentation) specimens were tested at each indentation angle, while CT analysis was performed on one representative specimen per angle. The results show that increasing the indentation angle had little effect on the peak indentation load but significantly increased the displacement at peak load and the total energy absorption. The CT analysis showed that the 30° indentation caused a wider and more distributed damage morphology compared to the localized damage pattern observed at 15°. Interestingly, the residual CAI strength of the 30° specimens was higher than that of the 15° specimens, which may be associated with the more distributed damage morphology and reduced severity of localized damage concentration. The progressive damage analysis using a homogenized ply-level Hashin progressive damage model implemented in Abaqus/Explicit is in good agreement with experimental results. These results provide insight into the mechanics of oblique contact damage in hybrid woven composites, which are important for damage-tolerant aerospace structural design.

## 1. Introduction

Fiber-reinforced polymer (FRP) composites, especially polymer matrix composites (PMCs), are widely used when high specific stiffness, high specific strength, and architectural design flexibility are required. Their behavior is not controlled by the reinforcement or matrix alone, but by the coordinated function of fibers, the polymer matrix, and the fiber–matrix interface. Fibers carry most of the applied load, the polymer matrix binds the architecture and transfers stress between fibers, and the fiber–matrix interface governs how efficiently load is transmitted and how damage is initiated and contained. As a result, composite performance at the structural scale is inseparable from constituent-level interactions, and the interface remains a central design consideration for achieving reliable and damage-tolerant response [1,2].

These advantages are especially valuable in aerospace structures, where mass reduction is directly associated with improved fuel efficiency, better payload efficiency, and lower environmental burden. Carbon fiber reinforced polymers (CFRPs) have therefore become key materials in aircraft structures and other lightweight systems, with the maturity of processing and certification practices enabling their use in major load-bearing components [3,4,5]. Yet the broader deployment of CFRPs is still shaped by two persistent constraints. The first is cost, since carbon fiber remains significantly more expensive than many alternative reinforcements, and precursor-related expenses continue to dominate overall material cost. The second is structural vulnerability under localized out-of-plane loading, where internal damage can accumulate even when the outer surface shows limited visible evidence [6].

This damage-sensitivity problem is commonly discussed in the framework of barely visible impact damage (BVID). Under low-velocity impact or indentation-type loading, laminated composites can develop interacting damage modes such as matrix cracking, inter-ply delamination, and fiber fracture, with the exact hierarchy depending on constituent properties, laminate architecture, and loading conditions [7,8]. Because much of this damage may remain hidden beneath the laminate surface, visual inspection alone is often inadequate for structural screening, and nondestructive testing (NDT) methods such as ultrasonic inspection, X-ray-based inspection, and other advanced diagnostic techniques are essential for damage detection and characterization [9]. More broadly, the resistance and tolerance of composite laminates to impact-related damage are known to depend strongly on resin toughness, fiber architecture, and hybrid configuration, the reason why material layout is treated as a damage-control variable rather than a purely manufacturing choice [10].

The importance of BVID becomes more pronounced when the damaged laminate is subsequently subjected to compression. In that case, residual strength is not governed only by the original indentation or impact event, but by the interaction between existing delamination, local instability, sub-laminate buckling, and further damage propagation during compressive loading. Compression after impact studies consistently shows that apparently modest damage can trigger substantial losses in compressive capacity, and that stacking sequence, material combination, and damage morphology strongly influence the onset of local buckling and the progression from internal damage to final collapse [11,12]. For this reason, composite damage-tolerance research increasingly treats the initial damage event and the residual compression stage as a coupled problem.

One widely used route to improve this coupled response is hybridization. By combining different fibers within a common matrix, hybrid laminates seek to balance stiffness, strength, strain-to-failure, cost, and damage tolerance more effectively than single-fiber systems. Carbon–glass hybrids are especially attractive because carbon fibers provide high stiffness and strength, while glass fibers contribute greater extensibility, lower cost, and improved tolerance to certain damage modes [13,14]. However, hybrid performance does not arise automatically from mixing fibers. It depends strongly on hybrid type, ply placement, and through-thickness arrangement, with interlayer architecture and stacking sequence affecting contact response, stress redistribution, and the development of delamination and fracture [15,16].

For woven composites, these issues become even more architecture-sensitive. The mechanical response depends not only on constituent selection but also on textile features such as warp and weft arrangement, weave pattern, yarn crimp, undulation, and interlacing frequency, all of which influence stiffness, stress concentration, and damage localization [17]. Because woven laminates possess a hierarchical geometry, their behavior is often interpreted across micro-, meso-, and macro-scales: fiber, matrix, and interface at the microscale; yarn path and tow interaction at the mesoscale; and lamina or laminate response at the macroscale. This multiscale perspective is particularly relevant for concentrated loading problems, where local architecture can govern the onset of damage that later controls global residual strength [18]. Most damage-tolerance studies still emphasize normal transverse loading, even though real service events are often oblique rather than perfectly perpendicular. Oblique contact introduces a tangential load component and frictional interaction in addition to the normal component, producing less symmetric stress transfer and potentially altering both the location and shape of damage. Recent work on oblique low-velocity impact indicates that increasing impact angle can change delamination patterns, redistribute subsurface damage, and modify energy absorption behavior, highlighting the need for systematic angle-dependent studies rather than assuming that normal loading alone is representative [19].

Quasi-static indentation (QSI) is well suited to this purpose because it provides a controlled means of studying concentrated out-of-plane loading while enabling stable acquisition of force–displacement data and repeatable observation of damage development. At the same time, its relationship to dynamic impact must be treated carefully: quasi-static loading can reproduce important aspects of damage progression, but loading rate and inertial effects still distinguish indentation from true impact response [20]. Even so, comparative studies show that QSI can be an effective approximation for certain low-velocity loading scenarios, particularly when combined with inspection and full-field measurement techniques that clarify how surface and internal damage evolve [21,22].

Against this background, the present study examines woven carbon–glass hybrid laminates under multi-angle quasi-static indentation using a hemispherical indenter at 15° and 30°, followed by compression after indentation (CAI) testing to assess residual compressive behavior. Residual compression performance is generally considered more critical than residual tensile performance in aerospace composite structures because impact-induced delamination remains stable under tensile loading but can rapidly propagate under compressive loading through local buckling and sub-laminate instability. Consequently, CAI testing has become the standard method for evaluating the damage tolerance of laminated composites. The analysis focuses on load–displacement response, energy absorption, and damage initiation and propagation during QSI, and on the resulting changes in compressive strength and stiffness during CAI. Because detailed specimen dimensions, loading-rate values, and units are not specified in this introductory summary, the emphasis here is on the governing mechanics and study rationale rather than test-specific magnitudes. This combined experimental framework is consistent with recent work showing that paired QSI and residual compression assessment can effectively connect damage morphology to structural performance in hybrid woven laminates [23].

To support interpretation of the experiments and extend the analysis beyond the tested configurations, the study also employs finite element modeling in Abaqus/Explicit. Predicting the residual compressive strength of damaged composite laminates requires consideration of the damage generated during indentation and its subsequent evolution under compressive loading. Liu et al. investigated the numerical prediction of compression-after-impact strength in damage-tolerant hybrid unidirectional/woven carbon-fiber composite laminates, demonstrating the relevance of progressive damage modeling to the residual performance of hybrid laminate systems [24]. Explicit-dynamics formulations combined with Hashin-based progressive damage criteria have also been used to reproduce global contact responses and effective intralaminar damage evolution in laminated composites [25]. However, Hashin-based intralaminar damage models do not explicitly represent interlaminar delamination unless a separate interface-damage formulation is introduced [25]. The constitutive basis for such simulations is provided by progressive damage modeling. The Hashin framework remains a foundational reference for distinguishing fiber tension, fiber compression, matrix tension, and matrix compression failure modes in fiber composites [26]. Physically based approaches such as those proposed by Puck and Schürmann further refine failure interpretation by accounting for matrix-dominated fracture planes and load-path effects [27]. Building on these foundations, continuum damage models have been developed to capture anisotropic stiffness degradation and post-initiation softening in laminated composites, and more recent three-dimensional (3D) Hashin-based implementations have improved representation of through-thickness stress effects and complex damage evolution under explicit analysis [28,29,30].

Within this setting, the present work aims to clarify how hybrid type, stacking sequence, and indentation angle affect the QSI response and the subsequent CAI performance of woven hybrid laminates, while using progressive damage simulation to support mechanism-based interpretation.

## 2. Materials and Methods

### 2.1. Fabrication of Composites

**Figure 1:**
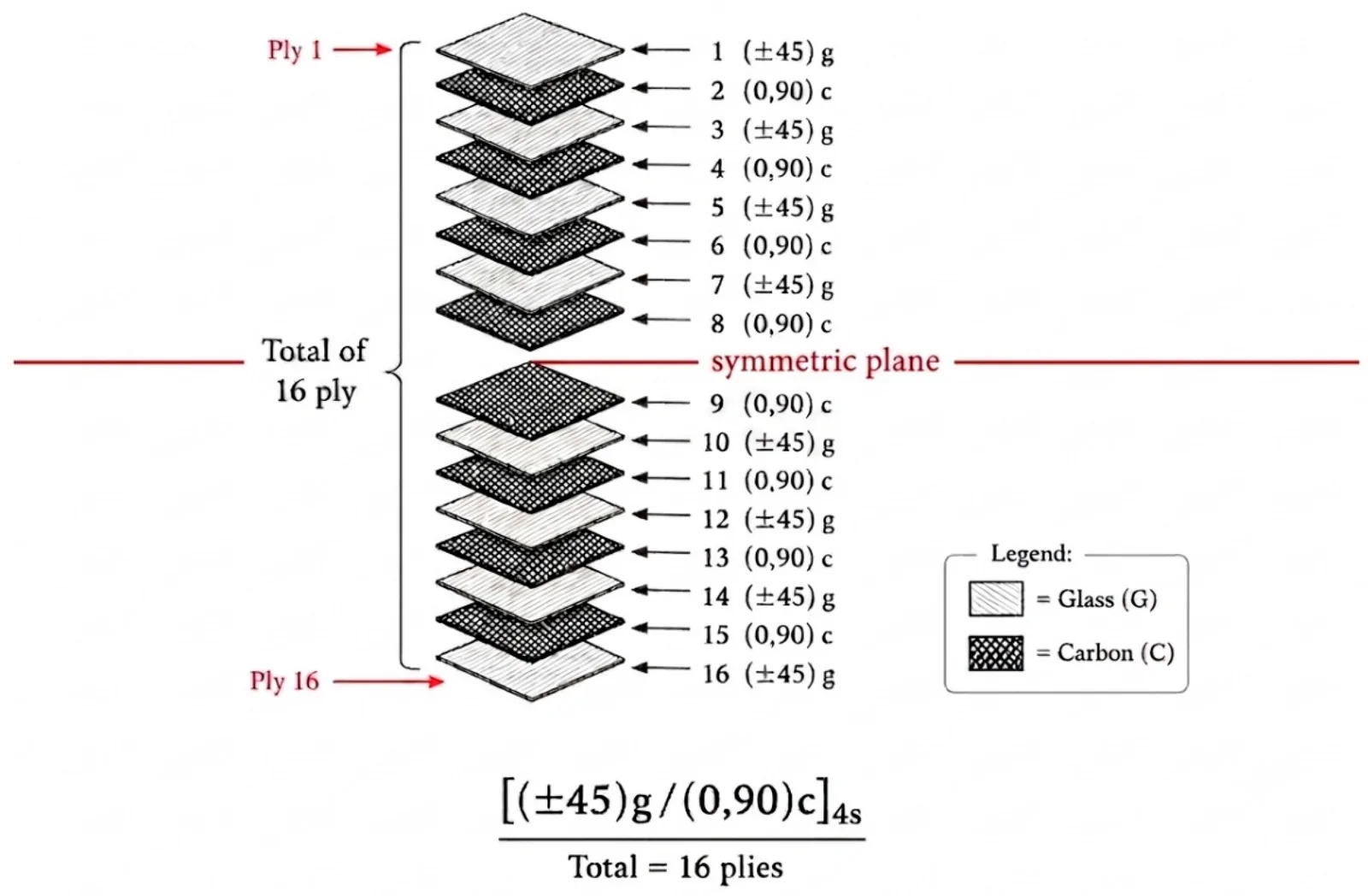
Stacking sequences of hybrid woven composite laminates [23].

Hybrid woven composite laminates were fabricated using plain-weave carbon fiber and plain-weave E-glass fiber fabrics with an epoxy resin, Epikote E51-618. The plain-weave fabrics were chosen for their balanced in-plane properties and improved structural stability compared to unidirectional laminates. Hybridization was done by combining carbon and glass plies into the laminate stacking sequence of [(±45)_g_/(0,90)_c_]_4S_, as shown in Figure 1. In the figure, g (G) and c (C) represent glass and carbon layers, respectively. The notation GC identifies the glass–carbon hybrid woven laminate. Individual indentation specimens were identified using the format indenter type–indentation angle–replicate number. The prefix “b” denotes the hemispherical indenter, the middle number denotes the prescribed indentation angle, and the final number identifies the experimental repeat. For example, b-15-3 represents the third specimen indented at 15° using the hemispherical indenter. The notation GC-15° refers collectively to the glass–carbon laminate specimens tested at 15°. The glass woven plies were rotated by 45° relative to the longitudinal specimen axis, such that their orthogonal warp and weft yarns were oriented at +45° and −45°, e.g., (±45)_g_. The carbon woven plies were aligned with their warp and weft yarns along the 0° and 90° specimen directions, e.g., (0,90)_c_. Each ply had a nominal thickness of 0.2 mm, giving a total laminate thickness of 3.2 mm. Composite laminates were manufactured by hand lay-up and vacuum consolidation to reduce the void content and to achieve a uniform resin distribution.

The laminate consisted of 16 woven plies arranged in a symmetric and balanced configuration to reduce residual stresses and bending–twisting coupling. These configurations were generated to provide a comparative basis for the evaluation of the effects of hybridization and stacking sequence. The laminates were vacuum-bagged and cured at a room temperature of 25 °C for 24 h, followed by a post-cure heat treatment at 60 °C for 2 h to stabilize the epoxy matrix network after lay-up. After curing, the composite panels were cut into rectangular specimens of approximately 150 mm by 100 mm with a diamond cutting saw. The thickness measurements were taken by a digital micrometer at different locations to ensure the dimensional consistency among all the specimens.

For QSI testing, three specimens were tested for each indentation angle. For CAI testing, two indented specimens were tested for each indentation angle. Results are reported as mean ± standard deviation, where repeated measurements were available. Before mechanical testing, the specimens were visually inspected for any defects such as fiber waviness, resin-rich areas, or voids. Selected samples were also examined by ultrasonic inspection to verify structural integrity prior to indentation testing.

### 2.2. QSI Tests

**Figure 2:**
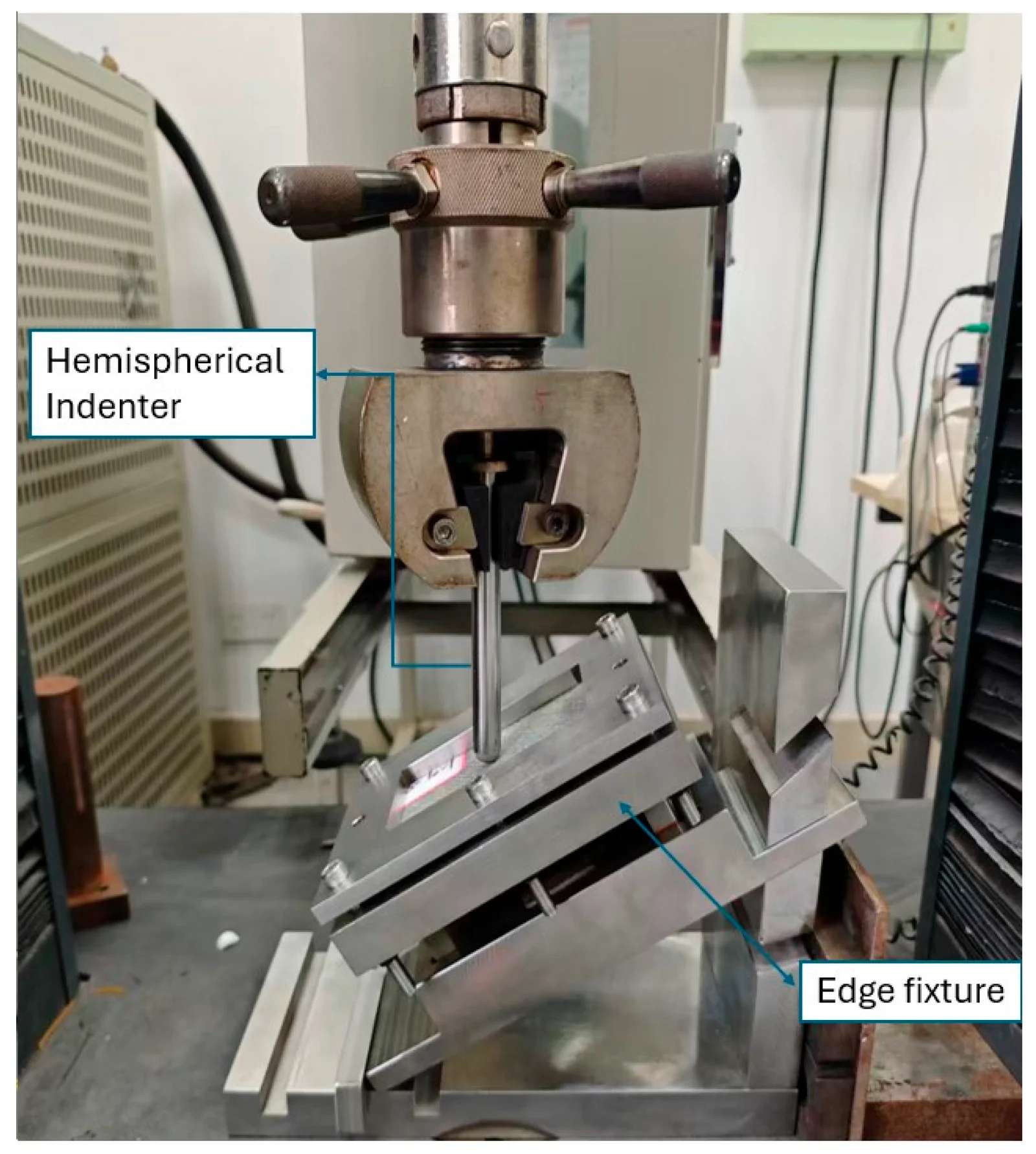
QSI test setup.

QSI tests were conducted to simulate localized out-of-plane loading conditions and generate controlled damage states within the composite laminates according to the ASTM D7136 standard [31]. This method allows accurate characterization of indentation resistance, force–displacement behavior, and damage evolution under concentrated loading. The tests were performed using a QSI test fixture with adjustable angles. A hemispherical steel indenter with a diameter of 16 mm was used to apply the indentation load. The specimen was clamped within a rigid support fixture containing a rectangular opening beneath the loading point to allow deformation during indentation, as shown in Figure 2.

To investigate the influence of loading orientation, indentation tests were performed at two different oblique angles relative to the laminate surface normal: 15° and 30°. These angles represent increasing levels of oblique loading, introducing both normal and tangential force components at contact. Previous studies have shown that oblique loading can significantly influence damage morphology and energy absorption characteristics in composite laminates. During testing, displacement-controlled loading was applied at a constant rate of 1 mm/min until the desired indentation depth or penetration was reached. The load and displacement data were continuously recorded to generate force–displacement curves for each specimen. The key parameters extracted from the indentation tests included: peak indentation load, indentation displacement at peak load, initial indentation stiffness, and energy absorption.

The absorbed energy was calculated as the area under the force–displacement curve using numerical integration. After indentation, the specimens were removed from the test fixture and visually examined to identify surface damage features such as indentation dents or fiber breakage. Internal damage characterization was conducted using CT-scan inspection. Selected specimens were inspected after the QSI, using an industrial micro-focus CT system (Diondo GmbH, Hattingen, Germany) at an X-ray voltage of 160 kV and a tube current of 110 μA. Total sampled frames were 4000. The reconstructed data had a matrix size of 3000 × 3000 and a voxel size of 0.037 mm. Reconstruction and damage analysis were performed using VGStudio MAX 3.4. A region of interest surrounding the indentation zone was selected, and the defect region was segmented from the intact material. Damage volume was calculated from the segmented defect voxels. Damage diameter was defined as the maximum in-plane extent of the segmented damaged region, while damage depth was measured from the original specimen surface to the deepest identified defect. This enables the detection of subsurface delamination and internal defects that are not visible on the surface. Non-destructive evaluation techniques are essential for identifying barely visible impact damage (BVID) commonly found in composite laminates [4].

### 2.3. CAI Tests

The CAI test was used to evaluate the residual compressive strength of the hybrid woven composite laminates after indentation damage. CAI testing is commonly used in aerospace structural assessment because composite laminates that appear only slightly damaged at the surface can experience significant reductions in compressive load-carrying capacity due to internal damage mechanisms such as delamination and matrix cracking with respect to the ASTM D7137 standard [32].

**Figure 3:**
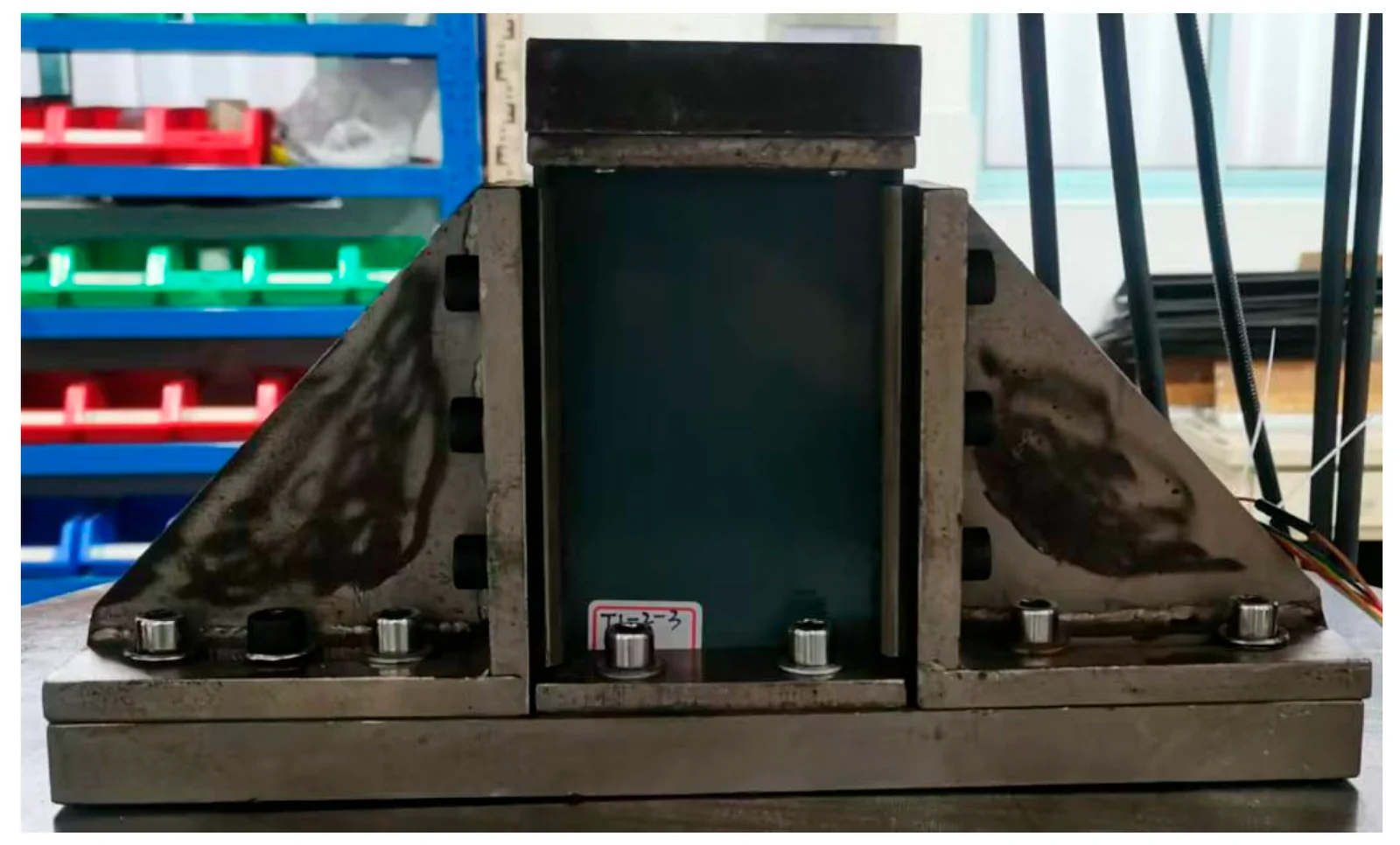
CAI test fixture with the specimen.

After completion of the quasi-static indentation tests, the indented specimens were compressed by means of a stabilized CAI test fixture, as shown in Figure 3. This fixture provides lateral support along the specimen edges and prevents global buckling during loading to ensure failure occurs mainly in the damaged region created during the indentation test. Specimens were carefully aligned in the fixture to avoid eccentric loading. The compressive loading was applied in displacement control at a crosshead speed of approximately 0.5 mm/min. The applied load and displacement were measured continuously throughout the test until the failure of the specimen.

The residual compressive strength was computed from the maximum load carried by the specimen during the compression test, taking into account the geometry of the specimen and the laminate thickness. This parameter is indicative of the laminate’s ability to maintain structural integrity after localized indentation damage. The post-failure observations showed damage modes such as fiber fracture, matrix cracking, and the propagation of interlaminar delamination, which was initiated during the indentation process. The experimental results of CAI were also used to evaluate the ability of the finite element model to predict the residual compressive behavior of the hybrid woven composite laminates.

### 2.4. Numerical Modeling

To complement the experimental investigation, finite element simulations were carried out to reproduce the QSI and CAI responses of the hybrid woven composite laminates. The numerical modeling gives a detailed analysis of stress distribution and damage evolution in composite laminates, which is difficult to observe directly in experiments. The simulations were carried out by employing the explicit dynamic solver based on the Abaqus 2024/Explicit software using the analysis and simulation method developed by Herath et al. [23], which is extensively utilized for the modeling of nonlinear contact problems and progressive damage in composite materials. The woven plies were modeled using an equivalent homogenized orthotropic lamina approach. The 3D Hashin criterion was used as a ply-level progressive damage criterion to distinguish fiber- and matrix-dominated failure modes within the homogenized woven layers. This approach is suitable for capturing the global force–displacement response and approximate damage localization.

The numerical analysis included two simulation steps. First, a quasi-static indentation simulation was performed on the laminate to introduce indentation damage. The damaged configuration thus obtained was utilized as the initial state of the CAI simulation to estimate the residual compressive strength of the laminate plate. The composite laminate was modeled as a 3D structure using layered composite elements, which are able to represent individual plies through a laminate thickness of 0.2 mm. The Composite Lay-up section was used to assign material orientation of each ply to reproduce the stacking sequence used in the experimental specimens. The hemispherical indenter was modeled as a rigid surface (R3D4) in order to reduce the computational cost and to avoid the unnecessary deformation of the loading tool. The rigid body method is often used during the simulation of the indentation because the stiffness of the indenter is much larger than that of the composite laminate. The laminate was discretized using eight-node continuum-shell elements with reduced integration (SC8R). A structured in-plane mesh with an approximate element size of 2 mm^2^ × 2 mm^2^ was employed, resulting in 3750 laminate elements. One continuum-shell element was used through the 3.2 mm laminate thickness.

**Figure 4:**
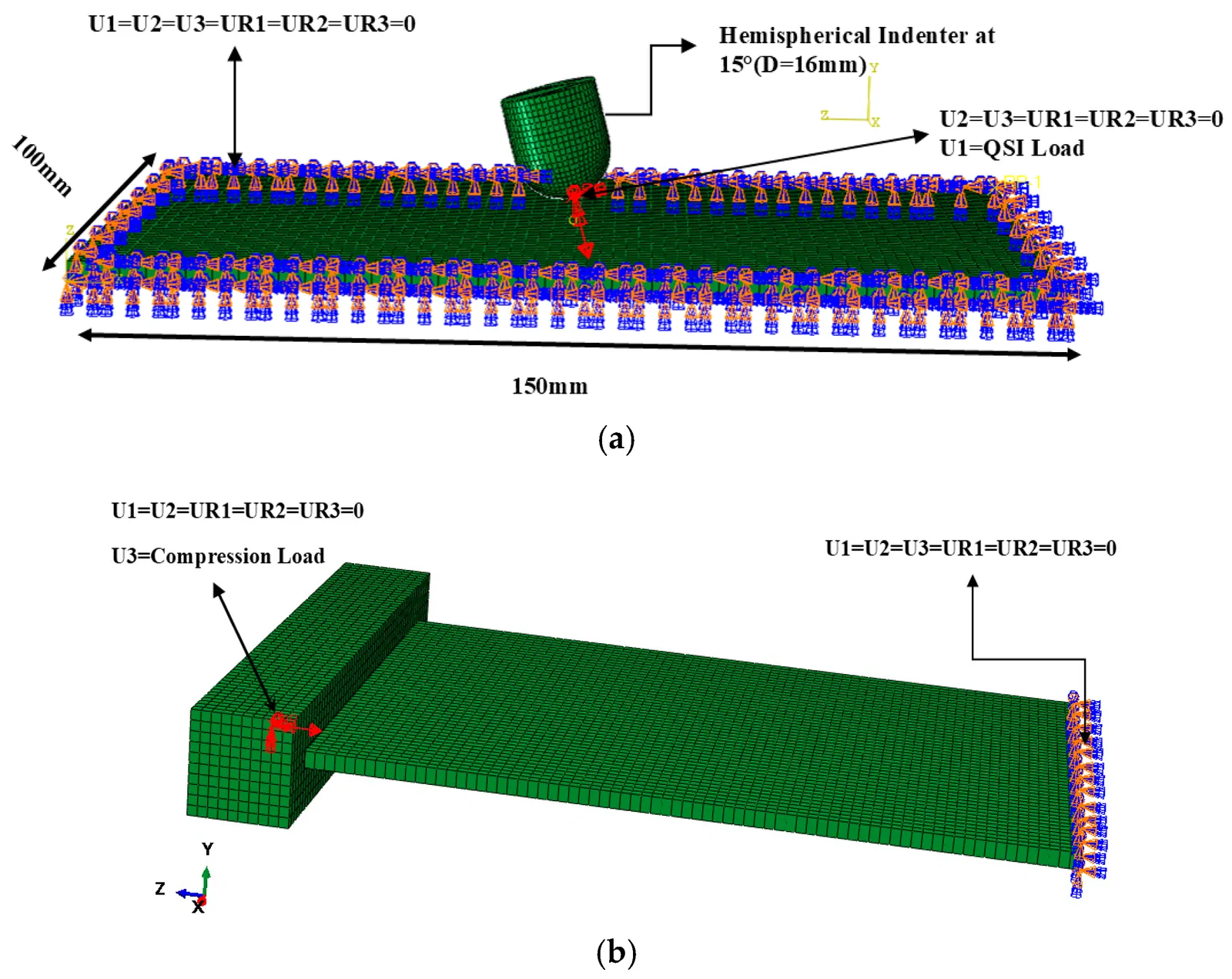
Boundary conditions of finite element models: (**a**) QSI; (**b**) CAI.

The boundary conditions were applied to mimic the experimental fixture used for the indentation testing. The laminate edges were constrained to mimic the clamping conditions of the support fixture (U1 = U2 = U3 = UR1 = UR2 = UR3 = 0), and only the indenter displacement U1 was prescribed to move in the loading direction (U2 = U3 = UR1 = UR2 = UR3 = 0). The indenter loading axis was inclined at 15° and 30° to the laminate surface normal to simulate oblique indentation. The configuration enables us to investigate the influence of the indentation angle on damage development in the composite laminate, as depicted in Figure 4.

Damage evolution in the composite laminates was modeled using a progressive damage framework. Intralaminar failure within the composite plies was predicted using the 3D Hashin failure criteria and contact between the rigid indenter and laminate was represented using a general contact setting with a friction coefficient of *μ* = 0.3, which distinguish different failure modes, including fiber tensile failure, fiber compressive failure, matrix tensile cracking, and matrix compressive crushing. The Hashin criterion was applied to each woven ply through an equivalent homogenized orthotropic-lamina representation. Accordingly, the fiber- and matrix-damage variables are interpreted as effective ply-level indicators rather than direct predictions of individual tow failure. Similar explicit-dynamics and Hashin-based approaches have been used to model the global impact response and intralaminar damage evolution of laminated composite structures [25].

The mechanical properties in Table 1 used in the finite element model were adopted from the previously published experimental and numerical characterization of comparable woven carbon/epoxy and glass/epoxy laminates reported by Herath et al. [23].

Once a failure criterion was satisfied, stiffness degradation was applied to the corresponding material direction to represent the reduction in load-carrying capacity caused by damage. The degradation process allowed gradual damage evolution rather than instantaneous element failure, enabling a realistic simulation of progressive damage in composite laminates. Interlaminar damage between plies was modeled using cohesive interaction properties, which enable delamination initiation and propagation. This is one of the dominant failure mechanisms in composite laminates that are subjected to transverse loading. The combined intralaminar and interlaminar damage modeling framework allowed the simulation to capture the interaction between matrix cracking, fiber breakage, and delamination growth during indentation and subsequent compression.

The quasi-static indentation process was simulated by applying displacement-controlled loading to the rigid hemispherical indenter. The loading rate was chosen to maintain the conditions close to static, and to reduce inertial effects in the explicit dynamic solver. During indentation, the contact force between the indenter and laminate was recorded to get the force–displacement data, which were later compared with experimental results for validation of the finite element model and analysis. The simulation captured several damage mechanisms typically observed during indentation of composite laminates, including matrix cracking beneath the indenter, fiber breakage in highly stressed regions, and delamination between adjacent plies.

After completion of the indentation simulation, the damaged laminate configuration was used as the initial condition for the CAI analysis. In this stage, the indenter was removed, and compressive displacement was applied to the laminate edges to reproduce the experimental CAI test conditions. Boundary conditions equivalent to the stabilized compression fixture were applied to prevent global buckling of the laminate. This guaranteed that the failure was concentrated in the region of indentation damage. The numerical model has been used to follow the evolution of damage variables and the deformation pattern of the laminate under compression loading. The residual compressive strength predicted by the simulation was calculated from the maximum load borne by the laminate in compression.

The simulation results indicated that the effect of indentation-induced delamination on the compressive failure was significant. The local instability of the damaged plies around the indentation zone was responsible for the fast propagation of the delamination and the resulting failure of the laminate.

## 3. Results and Discussion

### 3.1. QSI Behavior of Hybrid Laminates

**Figure 5:**
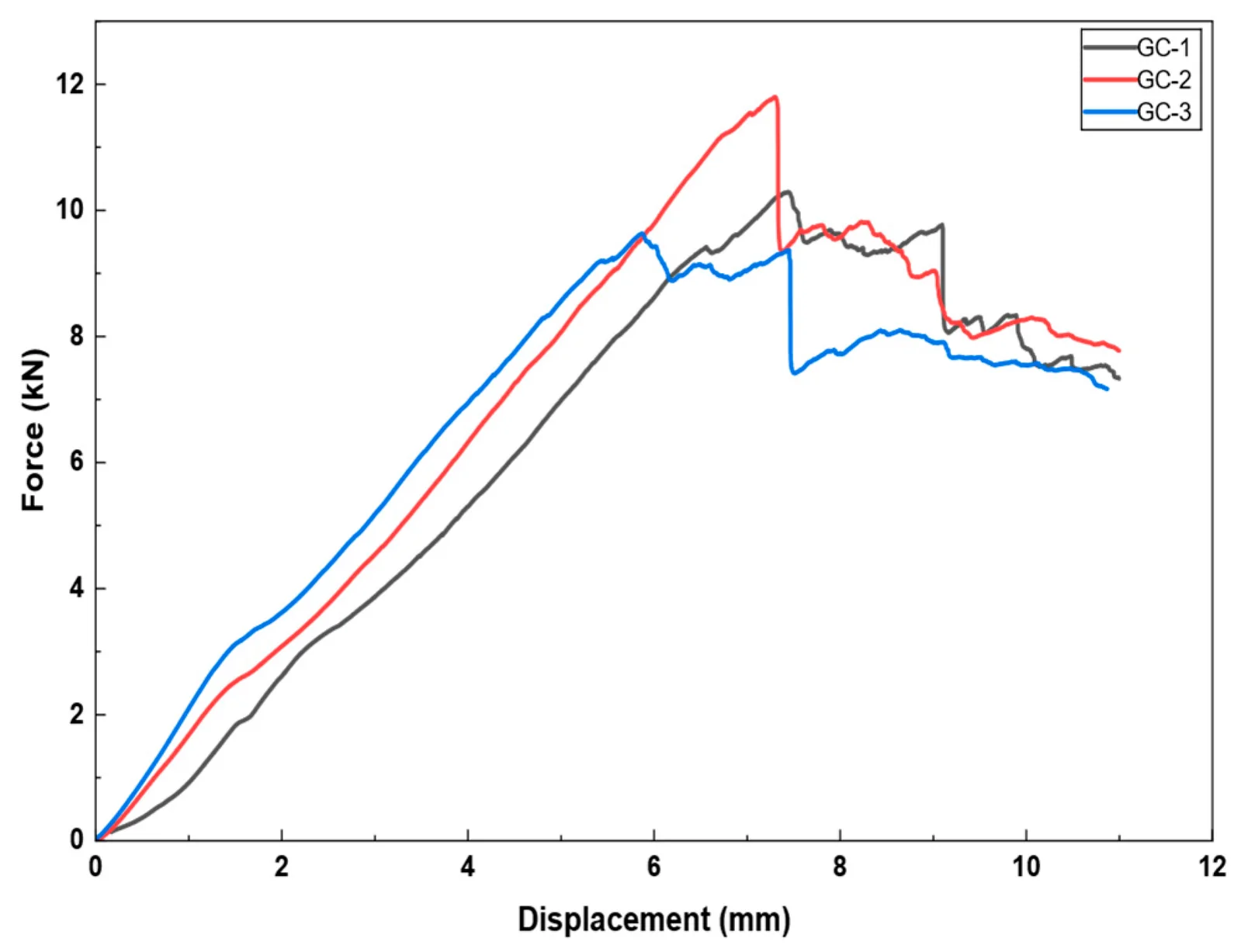
Force–displacement curves of GC laminates under 15° quasi-static indentation.

**Figure 6:**
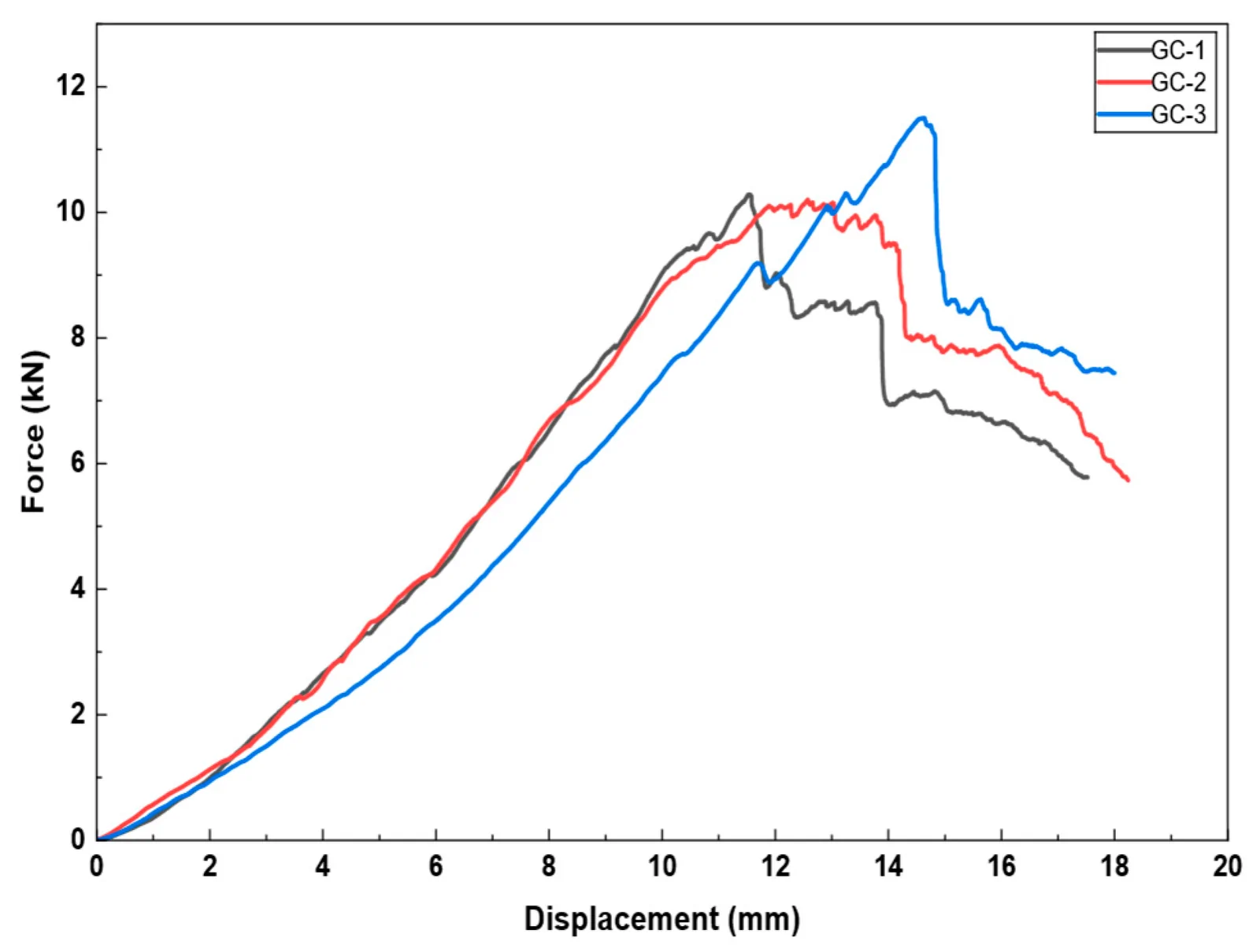
Force–displacement curves of GC laminates under 30° quasi-static indentation.

The QSI response of the GC hybrid woven composite laminate was investigated at oblique indentation angles of 15° and 30° using a hemispherical indenter. Three specimens were tested at each angle to evaluate experimental repeatability. The corresponding force–displacement curves are shown in Figure 5 and Figure 6. Both conditions exhibited three characteristic stages: an initial approximately linear loading stage, a nonlinear damage-development stage, and post-peak load degradation. The departure from linearity indicates progressive stiffness loss associated with the initiation and accumulation of internal damage. The three repeats at each angle produced similar overall curve shapes, although some variation was observed in the peak load and corresponding displacement.

The mean peak loads were 10.57 kN at 15° and 10.66 kN at 30°, representing a difference of less than 1%. In contrast, the displacement at peak load increased from 6.87 mm at 15° to 12.92 mm at 30°. Therefore, within the investigated oblique-angle range, the indentation angle influenced deformation more strongly than maximum load resistance. The larger deformation at 30° is attributed to the increased tangential contact component, which promotes frictional interaction, asymmetric stress transfer, and shear-assisted damage development. The principal QSI parameters are summarized in Table 2.

The displacement at peak load increased from 6.87 mm at 15° to 12.92 mm at 30°. The large increase indicates that the 30° specimens underwent more indentation deformation to reach the maximum resistance. The total energy absorbed also increased from 70.86 J for GC-15° to 102.44 J for GC-30°. Similarly, the energy absorbed up to peak load increased from 36.47 J at 15° to 63.16 J at 30°. Because the 30° tests reached larger final displacements, the total absorbed energy should not be interpreted as an equal-displacement comparison. Consequently, the energy absorbed up to peak load was emphasized as the more appropriate indicator of pre-peak energy dissipation. This value increased from 36.47 J at 15° to 63.16 J at 30°, indicating greater deformation and damage-related energy dissipation before maximum load under the 30° condition.

It should be emphasized that the comparison was conducted under identical loading conditions rather than equal absorbed energy. So, the increase in absorbed energy represents an inherent response of the laminate to increasing oblique loading angle.

**Figure 7:**
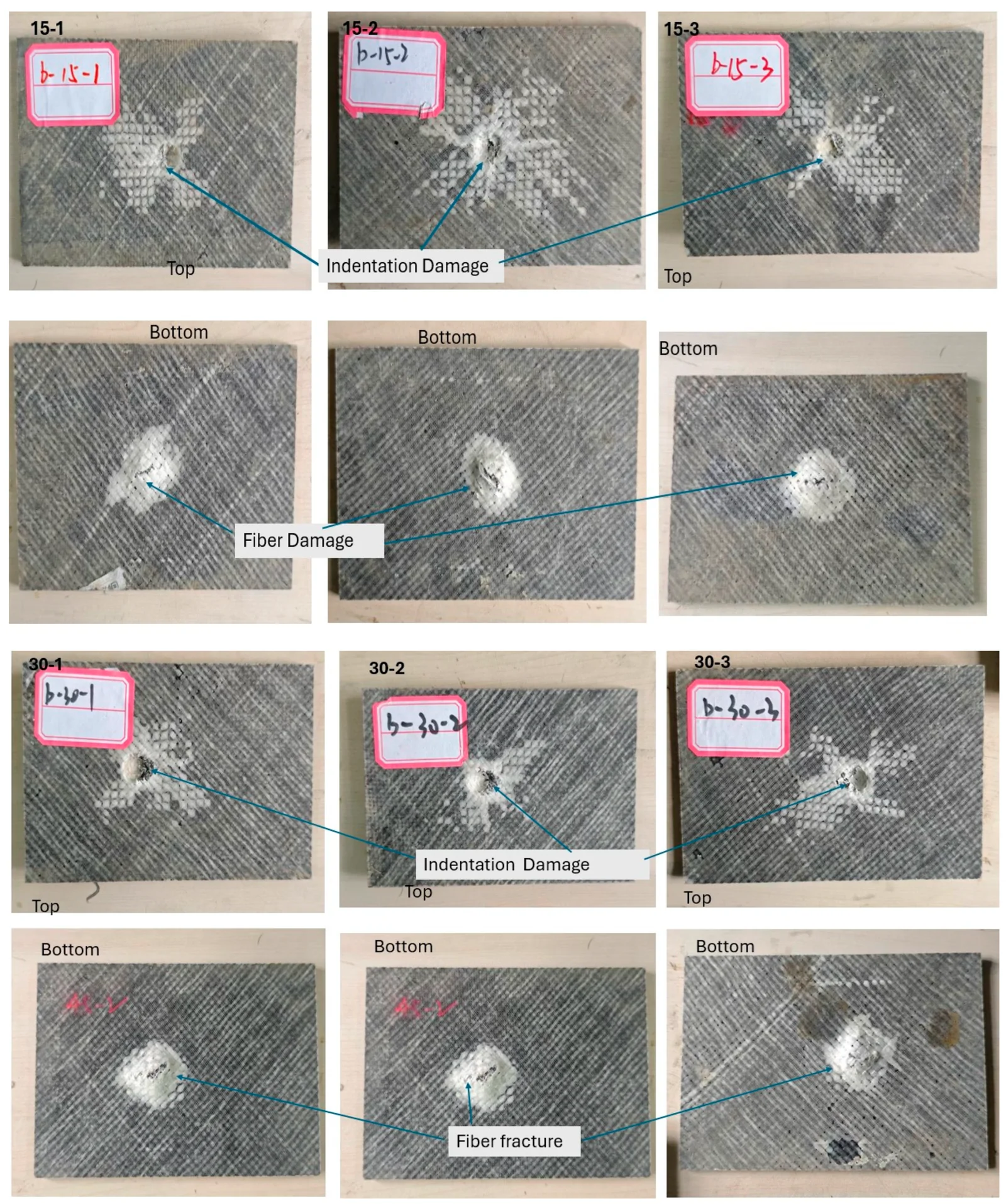
Surface damage morphology of specimens after QSI: top and bottom surfaces for 15° and 30° indentation.

The surface photographs after indentation are shown in Figure 7. Both top and bottom surfaces of the tested specimens were observed with clear indentation damage. For the 15° specimens, the top surface damage was primarily localized around the indentation region. Visible whitening, local matrix cracking, and indentation marks indicated localized crushing and matrix-dominated damage near the contact zone. Fiber damage and localized fiber fracture were observed on the bottom surface, which indicated that the indentation load produced through-thickness deformation and tensile damage on the opposite face of the laminate.

For the 30° specimens, the surface damage was more elongated and directional. The top surface showed a more distinct diagonal indentation mark, while the bottom surface showed more prominent fiber fracture and surface disruption in the vicinity of the damaged region. This indicated that the larger indentation angle favored asymmetric damage evolution. This behavior is consistent with oblique loading studies where the tangential force component alters the contact condition, and hence alters the damage morphology, compared to normal indentation or lower angle loading [19].

**Figure 8:**
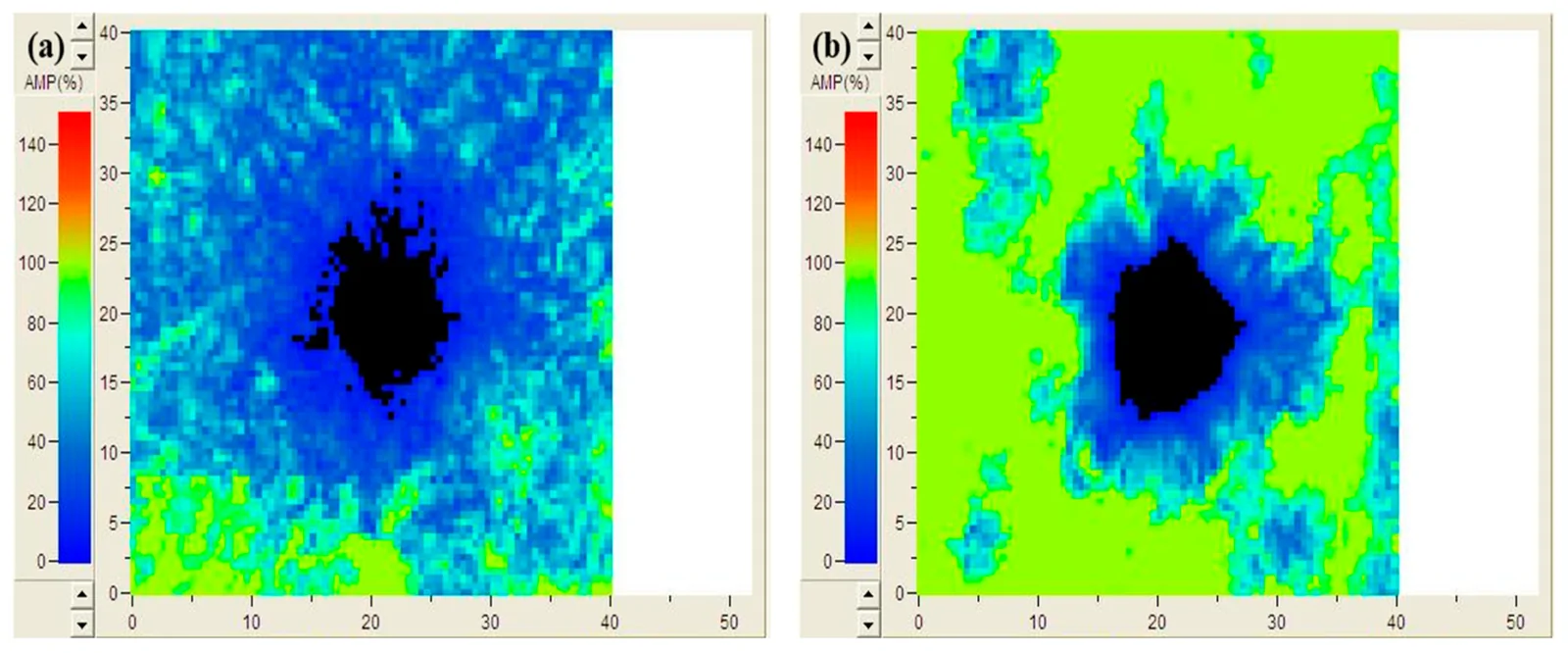
C-scan results after QSI: (**a**) 15° (b-15-1); (**b**) 30° (b-30-1).

The C-scan images in Figure 8 show a central damaged region around the indentation location for both 15° and 30° specimens. The dark central zone corresponds to the severely damaged indentation area, while the surrounding variations in color indicate changes in ultrasonic response caused by delamination, cracking, or internal discontinuities. In fact, the internal damage in composite laminates can be much larger than the visible surface dent, and the non-destructive inspection is important to understand the real extent of the indentation-induced damage.

For the GC-15° specimen, the C-scan damage region appeared more compact and concentrated around the indentation point. In comparison, the GC-30° specimen showed a wider and more distributed affected region around the indentation zone. The C-scan results confirm that the internal damage at 30° was wider and more asymmetric than that observed at 15°.

**Figure 9:**
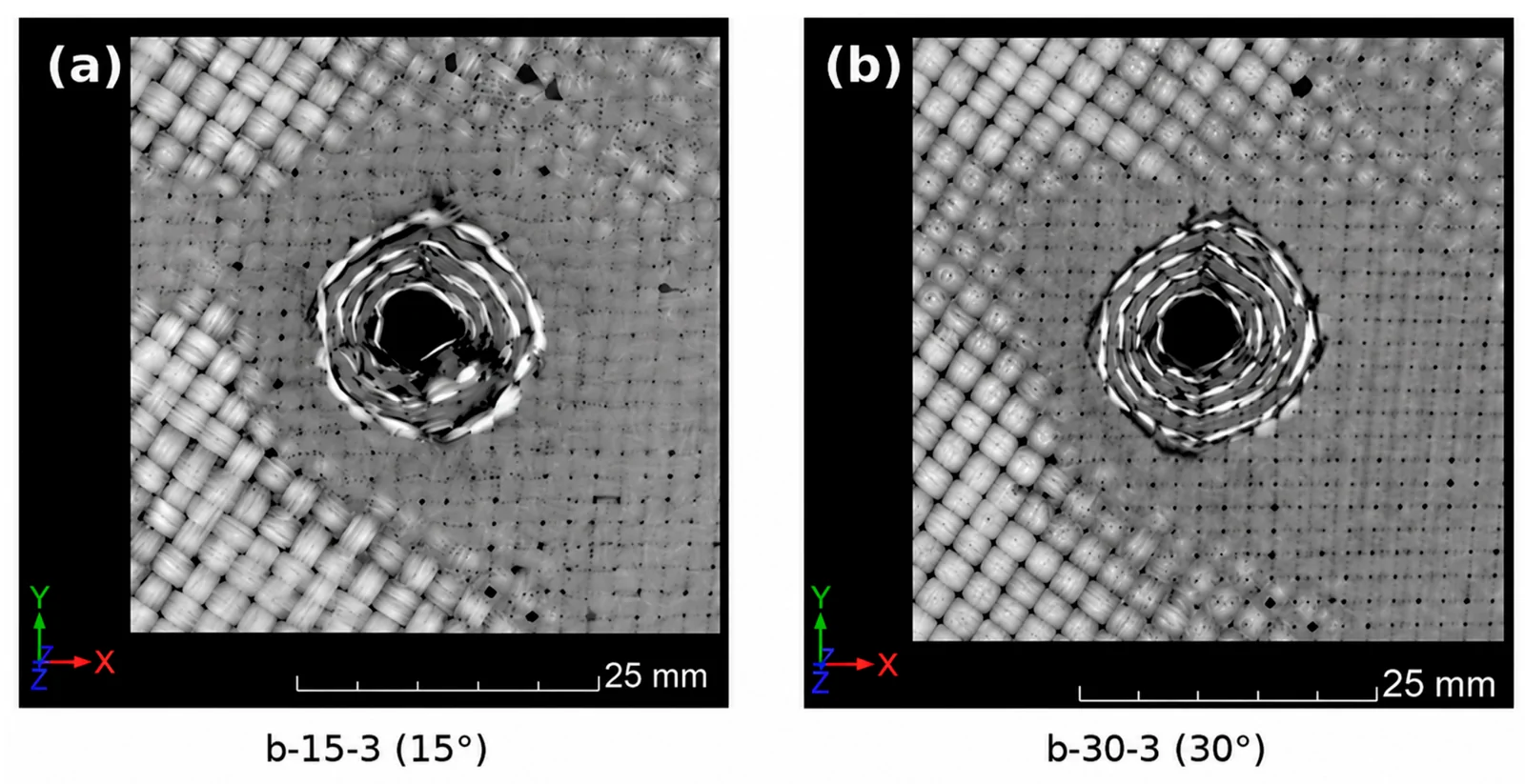
Top-view damage comparison: (**a**) 15° (b-15-3); (**b**) 30° (b-30-3).

**Figure 10:**
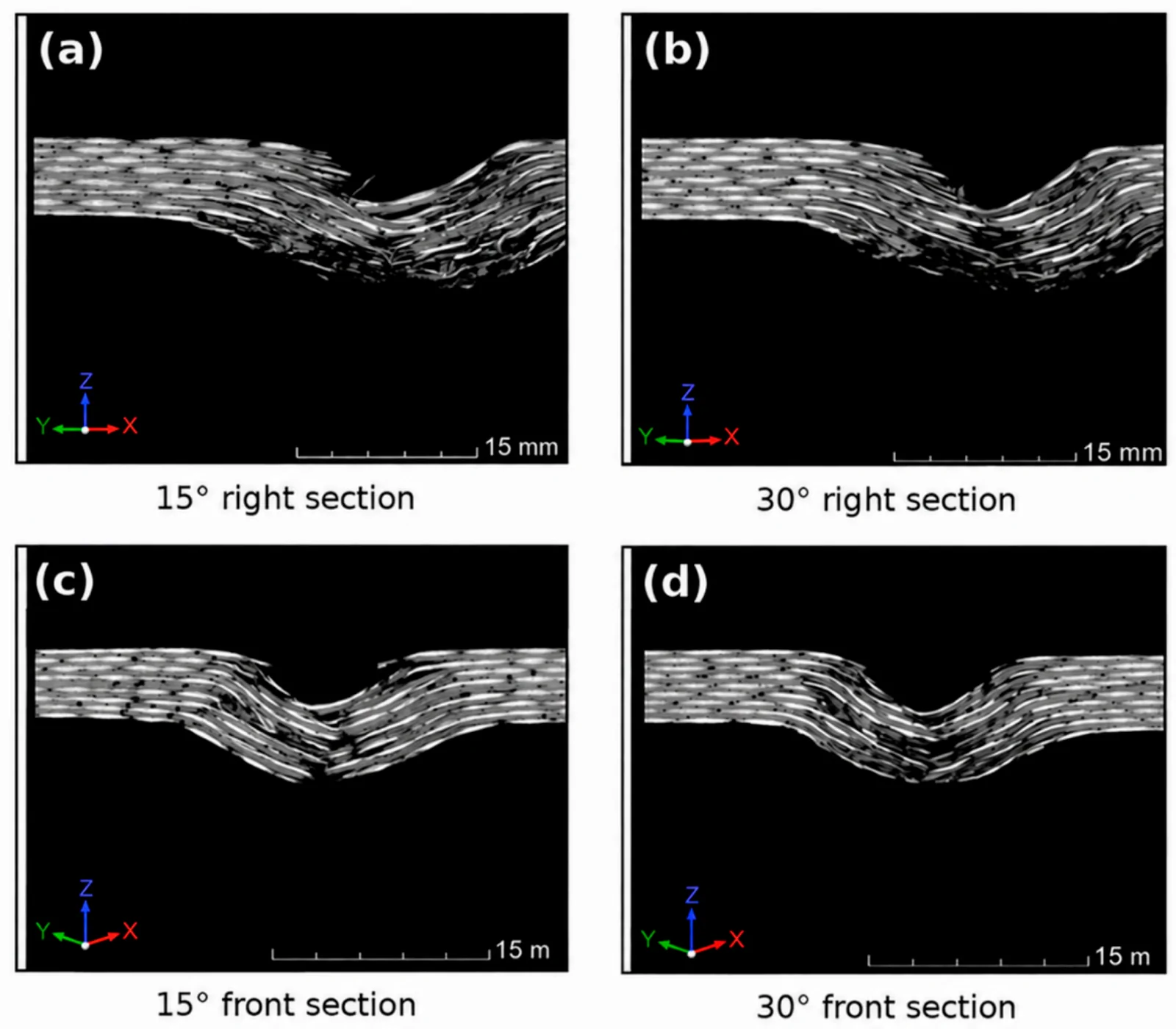
Cross-sectional damage comparison: (**a**) 15° right section; (**b**) 30° right section; (**c**) 15° front section; (**d**) 30° front section.

Table 3 summarizes the damage measurements for some selected specimens obtained by means of the computed tomography (CT). CT inspection was performed on 15-3 and 30-3 specimens, corresponding to the 15° and 30° cases, as shown in Figure 9 and Figure 10. Defect ratio for the b-15-3 was 6.87% while that for the b-30-3 was lower at 5.03%. However, the impact damage volume increased from 301.526 mm for the 15-3 specimen to 315.217 mm for the 30-3 specimen. The area of the damage surface was almost the same, 564.164 mm^2^ for 15-3 specimen and 561.433 mm^2^ for 30-3 specimen.

Damage diameter and the maximum damage depth were found to have a more distinct difference. The damage diameter increased from 16.791 mm at 15° to 20.366 mm at 30°, and the maximum damage depth increased from 4.359 mm to 4.594 mm. The results showed that the 30° indentation generated a wider and slightly deeper damaged region, in agreement with the higher displacement and energy absorption observed in the QSI load–displacement curves.

### 3.2. Numerical Analysis of QSI Tests

**Figure 11:**
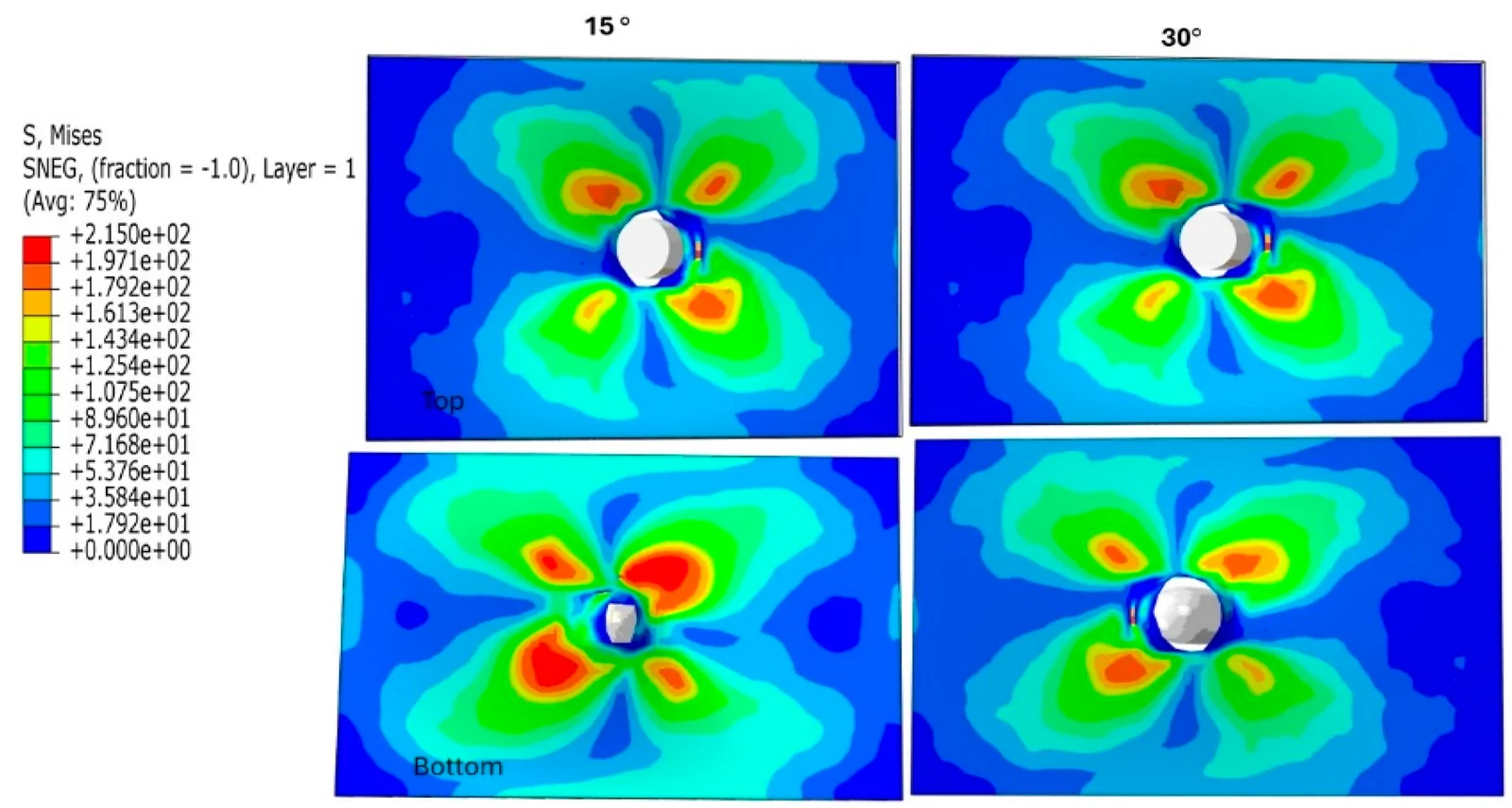
Simulated von Mises stress contours of GC laminates under 15° and 30° QSI.

The stress distribution and damage evolution of oblique indentation were further interpreted according to the numerical simulation results. The von Mises stress contours are illustrated in Figure 11. In both the 15° and 30° cases, high stress concentrations were observed around the indenter contact region. The stress field was not perfectly circular, but a multi-lobed distribution owing to the woven architecture and oblique contact direction. This indicated that the QSI response was controlled by both the local contact deformation and the anisotropic behavior of the woven laminate.

The matrix compression damage contours indicated that the damage was mainly localized around the region of indentation, where the local compressive and shear stresses were the maximum. For 15° QSI, the damage zone was relatively compact around the indenter. For the 30° case, the damage zone was more directional, indicating a more pronounced shear-assisted damage development. This is consistent with the experimental observation that the 30° indentation induced more elongated surface damage and larger displacement before the peak load.

**Figure 12:**
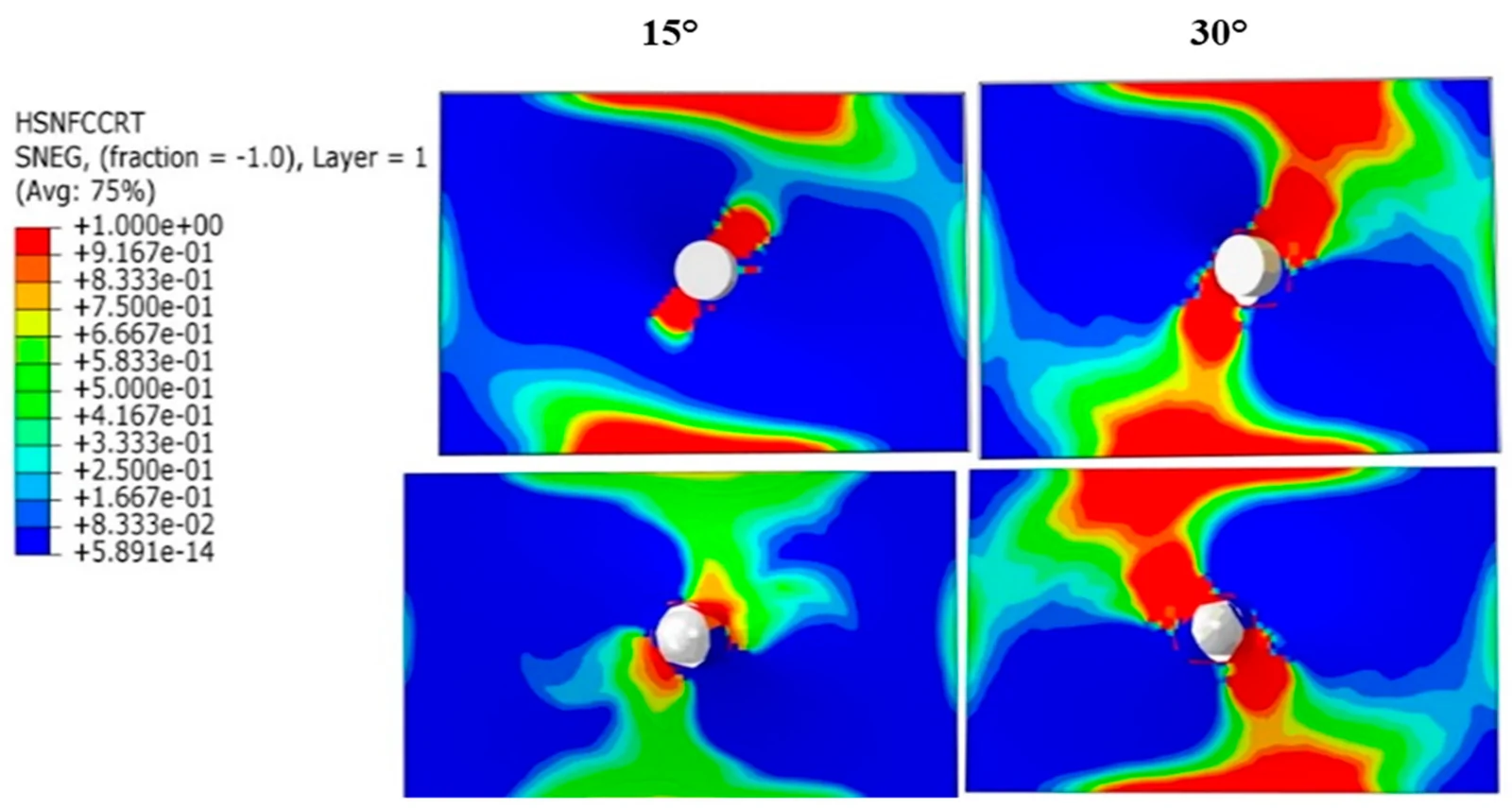
Simulated Hashin fiber compression damage contours under 15° and 30° QSI.

The contours of Hashin fiber compression damage are shown in Figure 12. For both angles, high fiber compression damage was found close to the indenter. The 30° case, however, showed more extensive damage to the fibers in the loading direction. This suggested that the higher oblique angle increased the tendency of directional crushing of the fibers and shear-assisted failure of the fibers. The numerical results are in agreement with the physical specimen observations, where fracture of the bottom surface fibers was more pronounced in the 30° case.

**Figure 13:**
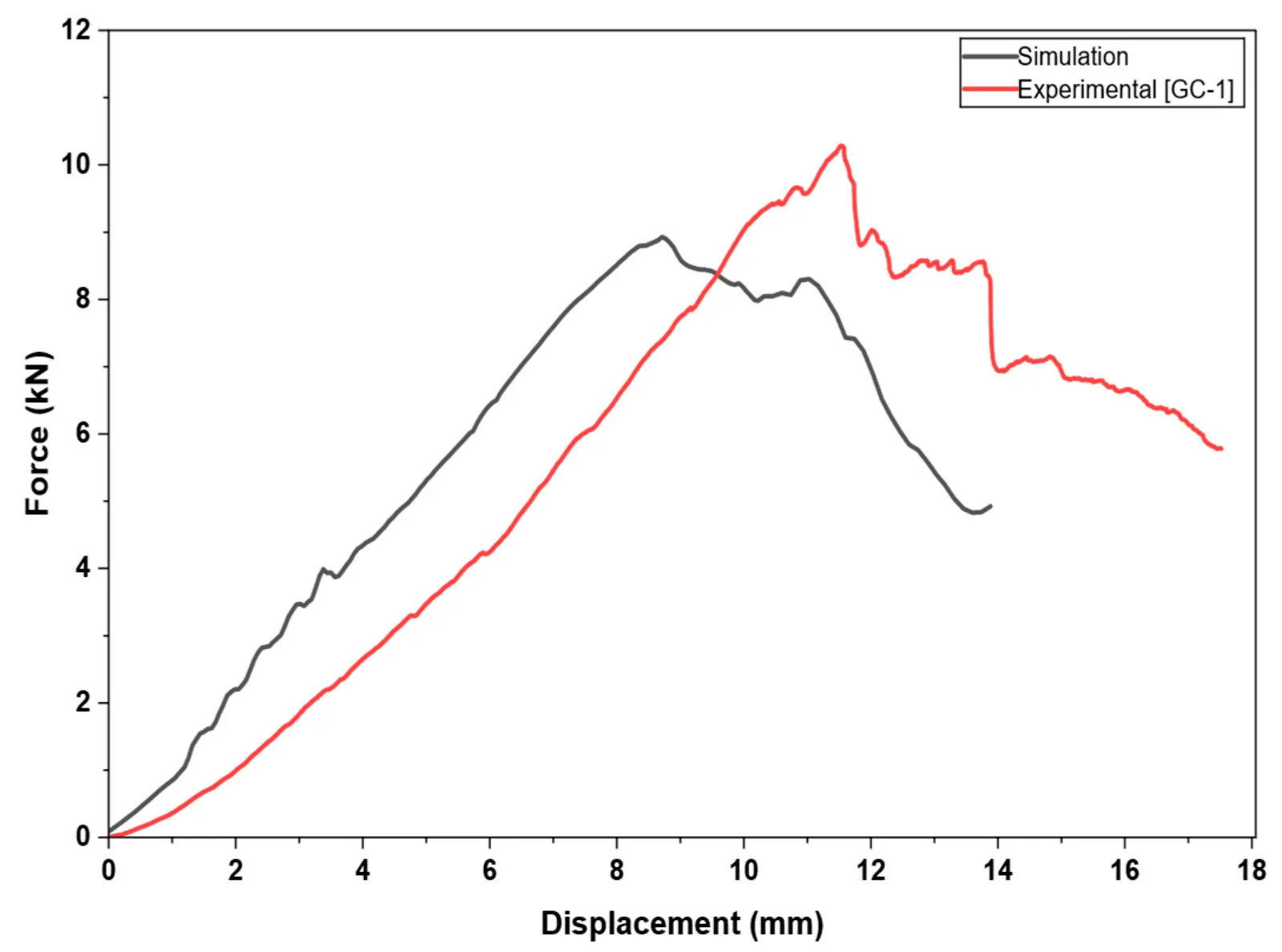
Experimental and numerical force–displacement comparison for GC-15° QSI tests.

**Figure 14:**
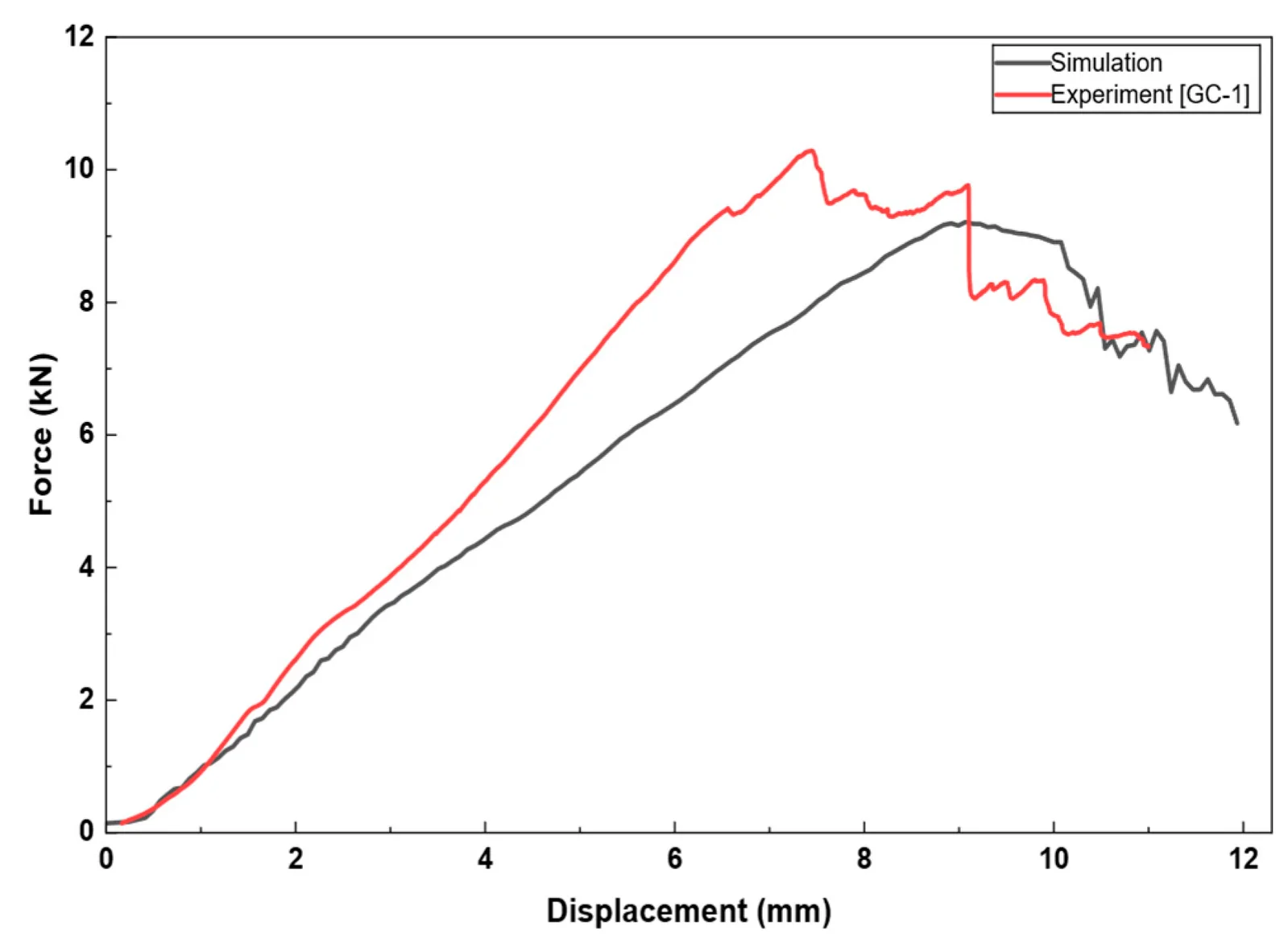
Experimental and numerical force–displacement comparison for GC-30° QSI tests.

The experimental and simulated force–displacement curves are compared in Figure 13 and Figure 14. The numerical model was able to capture the general trend of the experimental response for the 15° case. This included the initial increase in load, the nonlinear deformation stage, and the post-peak decrease in load. However, certain differences were observed in the stiffness and peak load, suggesting that the model did not fully reflect the experimental details.

For the 30° case, the difference between experiment and simulation was more significant, especially for the displacement at peak load. The experimental samples experienced a larger deformation before the maximum load, while the simulation predicted a faster load increase and an earlier peak response. This suggests that the numerical model could predict the general trend of damage, but underestimated the deformation capacity of the GC laminate under 30° oblique indentation.

Differences between experimental and simulated curves may be attributed to several modeling assumptions. Predicted response in the finite element model is highly sensitive to material damage parameters, interlaminar properties, contact friction, and boundary conditions. In oblique indentation, the interaction of indenter and laminate is more complicated than normal indentation, since the normal and tangential force components are included. Hence, the predicted force–displacement response can be strongly affected by small variations in the friction coefficient, the cohesive degradation law, or the stiffness reduction parameters. In addition, unavoidable experimental errors also cause the difference.

Despite these discrepancies, the numerical model was able to reproduce the main QSI behavior and damage locations. The simulation reproduced stress concentration around the indenter, matrix damage in the vicinity of the contact region, and fiber compression damage in the indentation zone. Therefore, the model is useful to interpret the experimental results but needs further calibration to improve the prediction accuracy, especially for the 30° case. It should be emphasized that limited sliding at the indenter laminate contact interface is an inherent feature of oblique indentation rather than an experimental artifact. The prescribed loading path generates both normal and tangential contact components, thereby promoting frictional interaction and localized sliding at the contact surface. In the finite element model, this behavior was represented through general contact with a Coulomb friction coefficient of *µ* = 0.3. This interface sliding is distinct from the rigid-body movement of the specimen within the fixture, which would represent an experimental error. During testing, specimen movement was minimized by seating the laminate within the rigid inclined support frame and mechanically restraining its perimeter using the surrounding metallic edge fixtures, while the central region remained exposed above the fixture opening to permit out-of-plane deformation. Accordingly, the wider and more directional damage morphology observed at 30° is attributed to the combined effects of normal indentation, tangential loading, frictional interaction, and localized contact sliding under the prescribed oblique loading condition.

Overall, the numerical model reproduced the principal experimental trends, including comparable peak indentation loads and a more directionally distributed damage pattern at 30°. The remaining discrepancies in curve shape and displacement response are attributed to the homogenized ply representation, contact assumptions, and experimental variability.

### 3.3. CAI Behavior of Hybrid Laminates

The CAI response is important as the compression loading can induce local buckling, delamination growth, matrix cracking, and fiber fracture, which can cause a reduction in the load-carrying capacity of laminates [23]. Hence, CAI tests were performed to investigate the residual compressive behavior of the GC hybrid woven composite laminates after oblique QSI.

**Figure 15:**
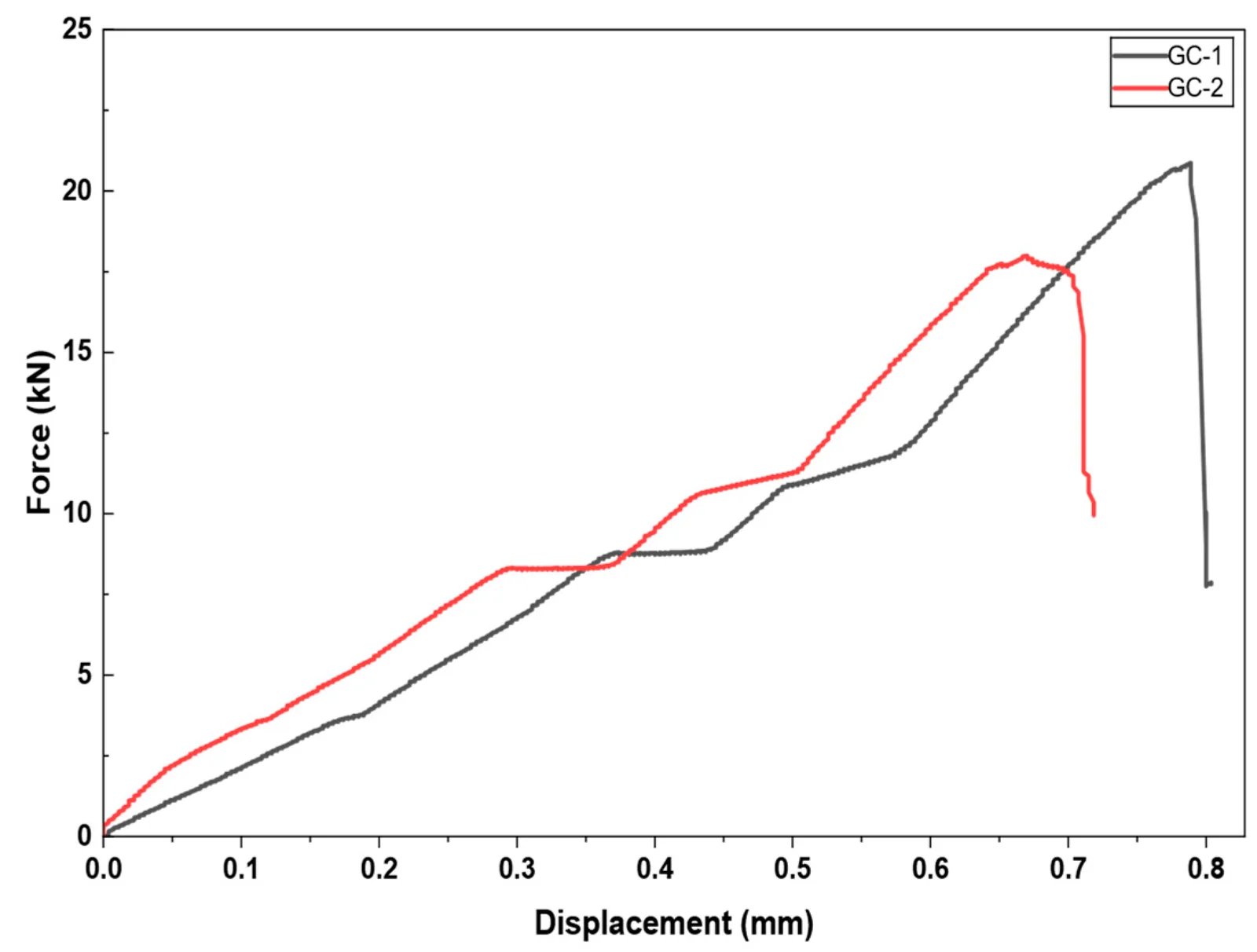
Experimental CAI load–displacement curves of GC laminates after 15° indentation.

**Figure 16:**
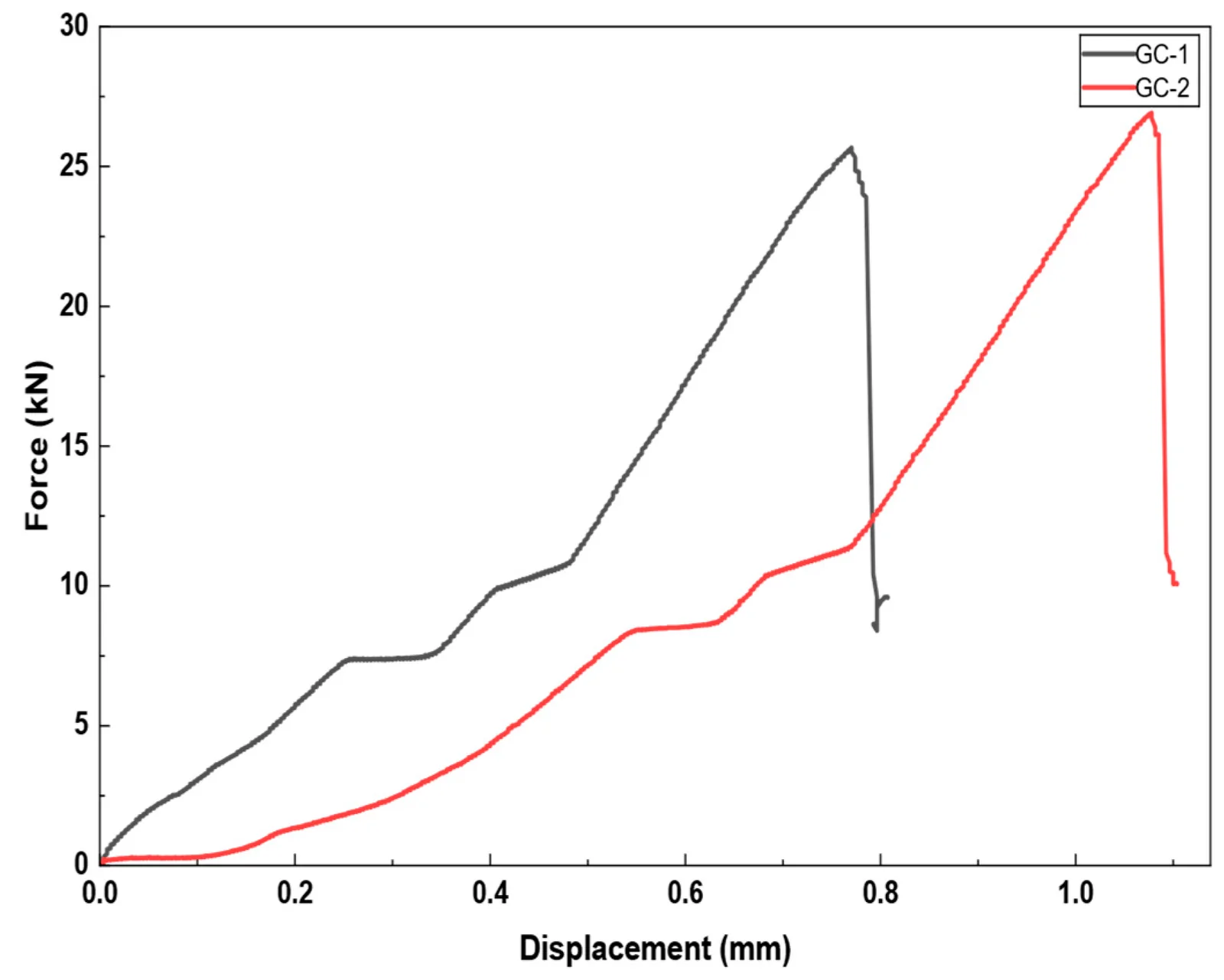
Experimental CAI load–displacement curves of GC laminates after 30° indentation.

The experimental CAI load–displacement curves of the 15° and 30° QSI tested specimens are presented in Figure 15 and Figure 16, respectively. For both angles of indentation, the curves increased almost linearly at the beginning, which indicates the elastic compressive response of the damaged laminate. With increasing compressive displacement, the curves started to deviate from linearity because of progressive degradation of stiffness due to the growth of local damage in the vicinity of the indentation zone. The dramatic drop of load is related to unstable damage propagation and compressive failure of the specimen.

The CAI experimental results for GC laminates are listed in Table 4. The maximum CAI loads for the 15° QSI tested specimens were 20.87 kN and 17.99 kN, with an average load of 19.43 kN. The corresponding average displacement at peak load was 0.730 mm. The mean residual compressive strength of the 15° QSI tested specimens was evaluated at 60.72 MPa. The maximum loads of CAI of 30° QSI tested specimens were 25.67 kN and 26.92 kN, respectively. The average peak load was 26.30 kN, and the average displacement at peak load was 0.924 mm. The corresponding residual compressive strength was 82.18 MPa. The residual compressive strength of 30° QSI tested specimens increased by approximately 35.36% compared with 15° QSI tested specimens.

This outcome suggests that the indentation angle significantly affected the post-indentation compressive performance of the laminate. The 30° QSI tested specimens absorbed more energy during QSI and had more deformation before the peak indentation load, but had a higher compressive capacity during CAI. This suggests that the damage produced at 30° QSI was more distributed and less critical to compressive collapse than that produced at the 15° QSI test. The contrary might be true for the 15° indentation, as it might have created more localized damage that acted as a more powerful stress concentration during compression.

**Figure 17:**
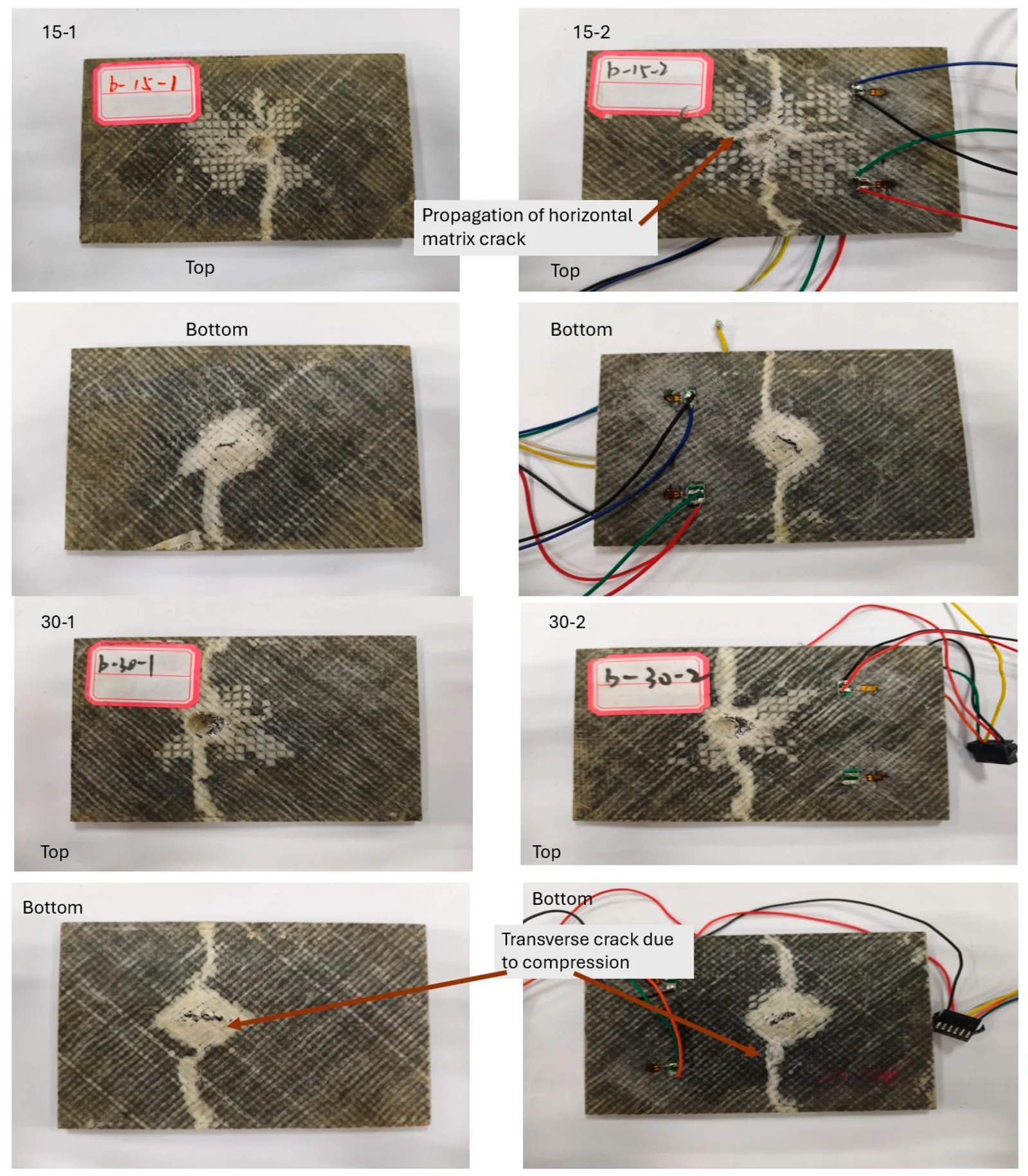
Post-CAI surface damage morphology of GC laminates after 15° and 30° indentation.

The post-CAI damage photographs are shown in Figure 17. For both indenter angles, the failure was mainly around the pre-damaged indentation region, confirming the fact that the QSI-induced damage was the critical location for the compressive failure. The damaged regions exhibited visible matrix cracking, fiber fracture, and crack propagation through the specimen surface.

For the 15° QSI tested specimens, the damage was more localized around the indentation region and propagated through the laminate as transverse cracks and fiber damage. The bottom surface showed clear evidence of fiber fracture and crack propagation from the indentation zone. This indicates that the localized indentation damage facilitated early instability and compressive failure under the compressive loading.

The damage pattern after CAI was more extended in the loading direction for the 30° QSI tested specimens. Surface imaging showed signs of horizontal propagation of matrix cracks and transverse cracking under compression. The cracks were initiated in the zone of indentation and propagated outwards. This implied that the pre-existing damage introduced by indentation resulted in a progressive compressive failure. However, this more extensive visible damage pattern in the 30° QSI tested specimens resulted in a higher compressive load capacity than the 15° QSI tested specimens.

This behavior can be attributed to the different indentation damage morphology developed during QSI. The 30° indentation created a wider and more distributed damage zone, while the 15° indentation produced more compact localized damage. Localized damage may serve as a more intense stress concentration and stimulate earlier sub-laminate instability under compression. Thus, the GC-15° samples failed at a lower compressive load, while the GC-30° samples exhibited higher residual strength due to a more distributed damage field. The CT measurements provide a quantitative context for the residual compression results. Compared with b-15-3, the b-30-3 specimen exhibited a 21.29% larger damage diameter and a 5.39% greater maximum damage depth, whereas damage volume increased by only 4.54% and damage surface area differed by less than 0.5%. At the same time, the average residual CAI strength increased by 35.36%. These measurements suggest that the principal difference between the two selected damage states was their spatial distribution rather than a large increase in total damaged volume. The wider 30° damage morphology may therefore have been less severe as a localized trigger for compressive collapse. Nevertheless, because CT was performed on only one selected specimen per angle, this interpretation is considered as supporting evidence rather than a statistically established relationship.

### 3.4. Numerical Analysis of CAI Tests

Finite element simulations were conducted to reproduce the CAI response of the indented GC laminates. The numerical model followed the experimental loading configuration, in which one side of the specimen was constrained while compressive displacement was applied along the longitudinal direction. The boundary condition in CAI simulation is shown in Figure 4.

**Figure 18:**
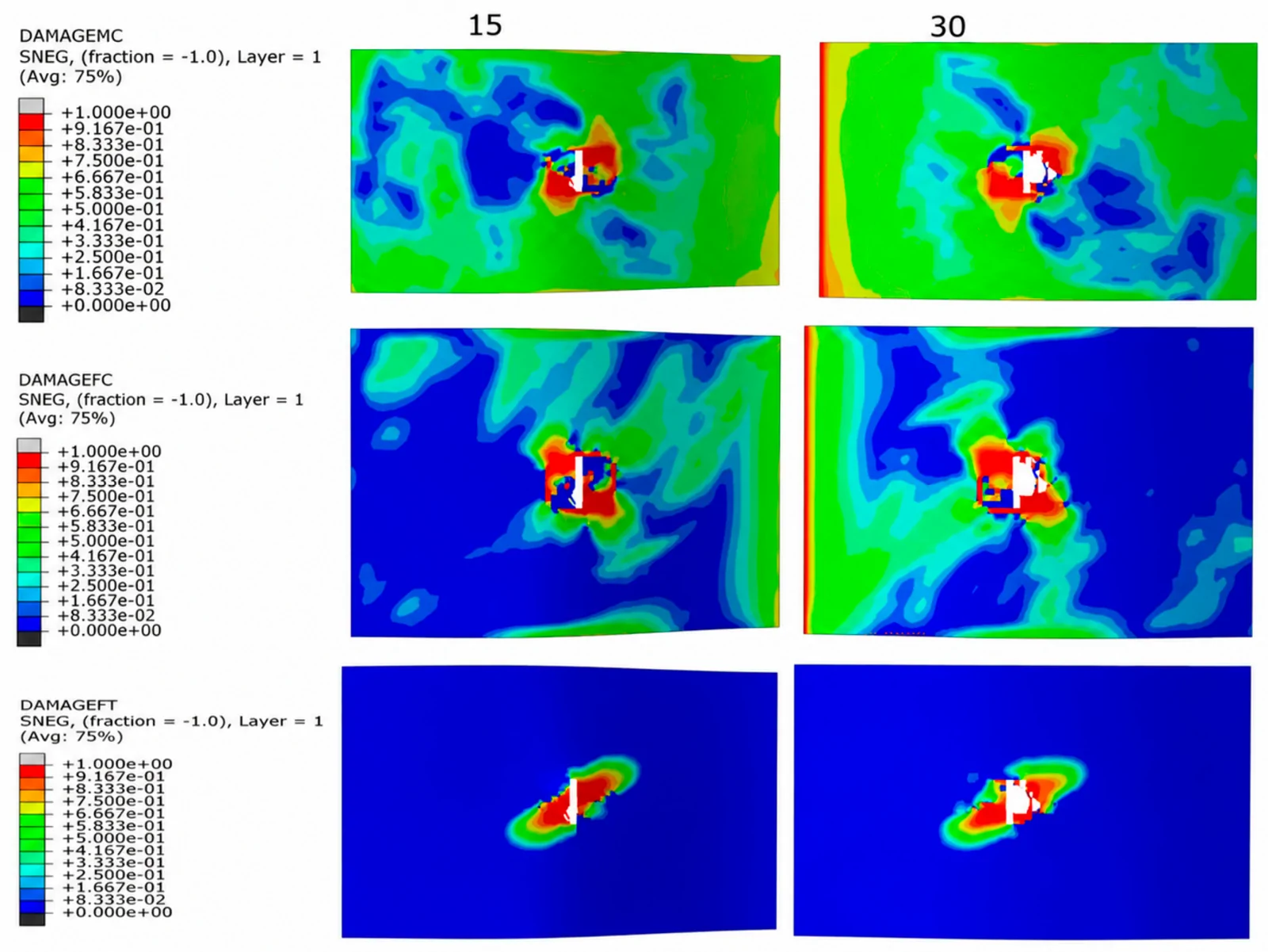
Simulated CAI damage contours showing matrix compression damage, fiber compression damage, and fiber tensile damage for 15° and 30° QSI tested specimens.

The numerical damage contours are shown in Figure 18. Matrix crushing damage, fiber crushing damage, and fiber tensile damage were localized around the indentation region, confirming that the pre-existing QSI damage dominated the subsequent compressive failure process. The maximum damage for the 15° and 30° cases was found to be near the indentation site, where the stress concentration and the local instability were most severe.

The matrix compression damage contours showed that the compressive damage appeared around the indentation region and propagated outward with the increase in compressive load. This suggested that the matrix played an important role in the initial stage of the CAI failure by facilitating local crushing and crack growth. The fiber compression damage contours indicated localized fiber failure near the indentation site, while the fiber tensile damage contours indicated that bending and local instability also caused tensile damage in selected regions of the laminate. The damage region in the 30° simulation was larger and more distributed than in the 15° case. This agrees with the experimental results where 30° specimens exhibited more extended crack propagation after CAI. The simulation supports the interpretation that the 30° indentation produced a more distributed damage field and the 15° indentation caused more localized damage that reduced the residual compressive capacity more strongly.

**Figure 19:**
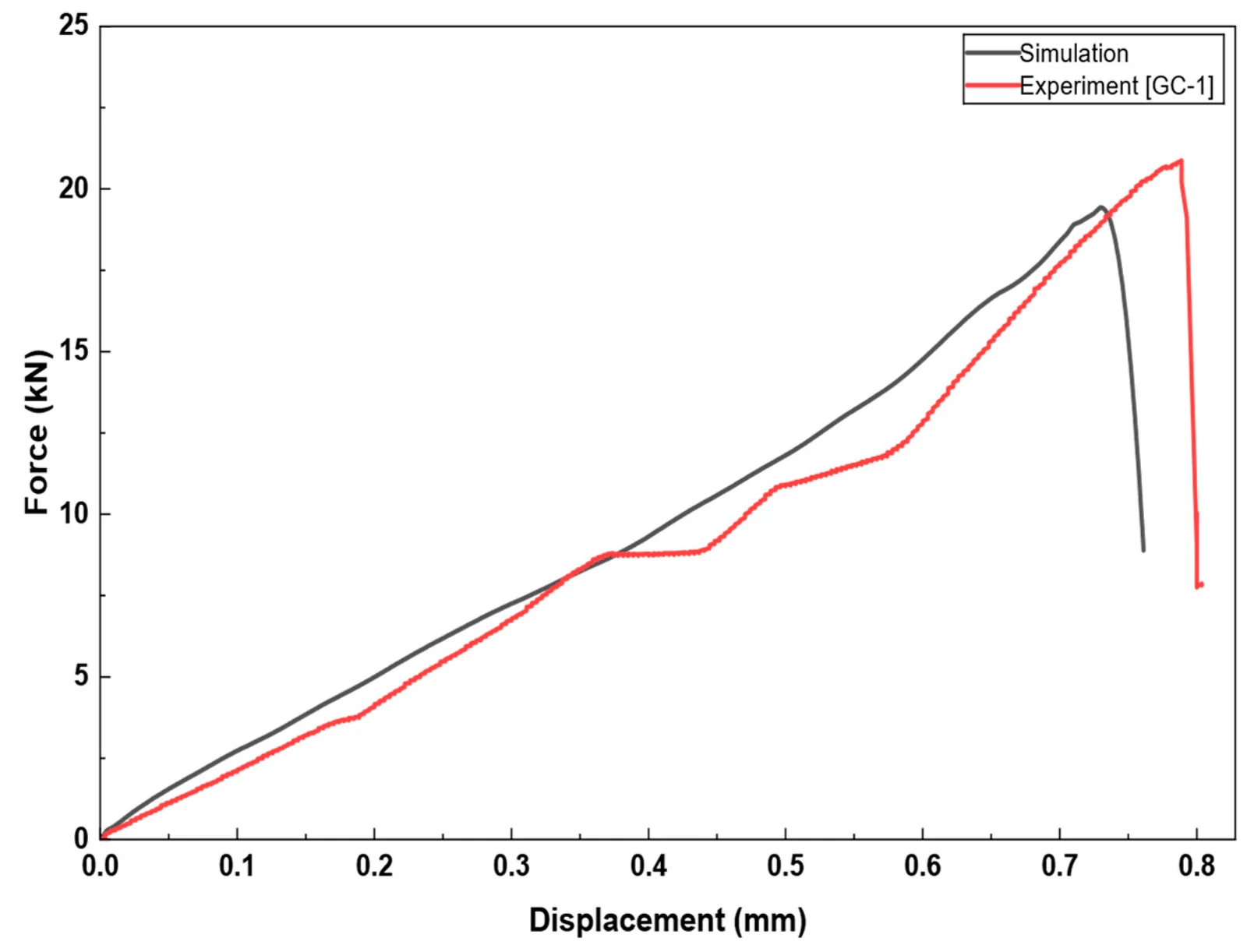
Experimental and numerical CAI load–displacement comparison for 15° QSI tested specimen.

**Figure 20:**
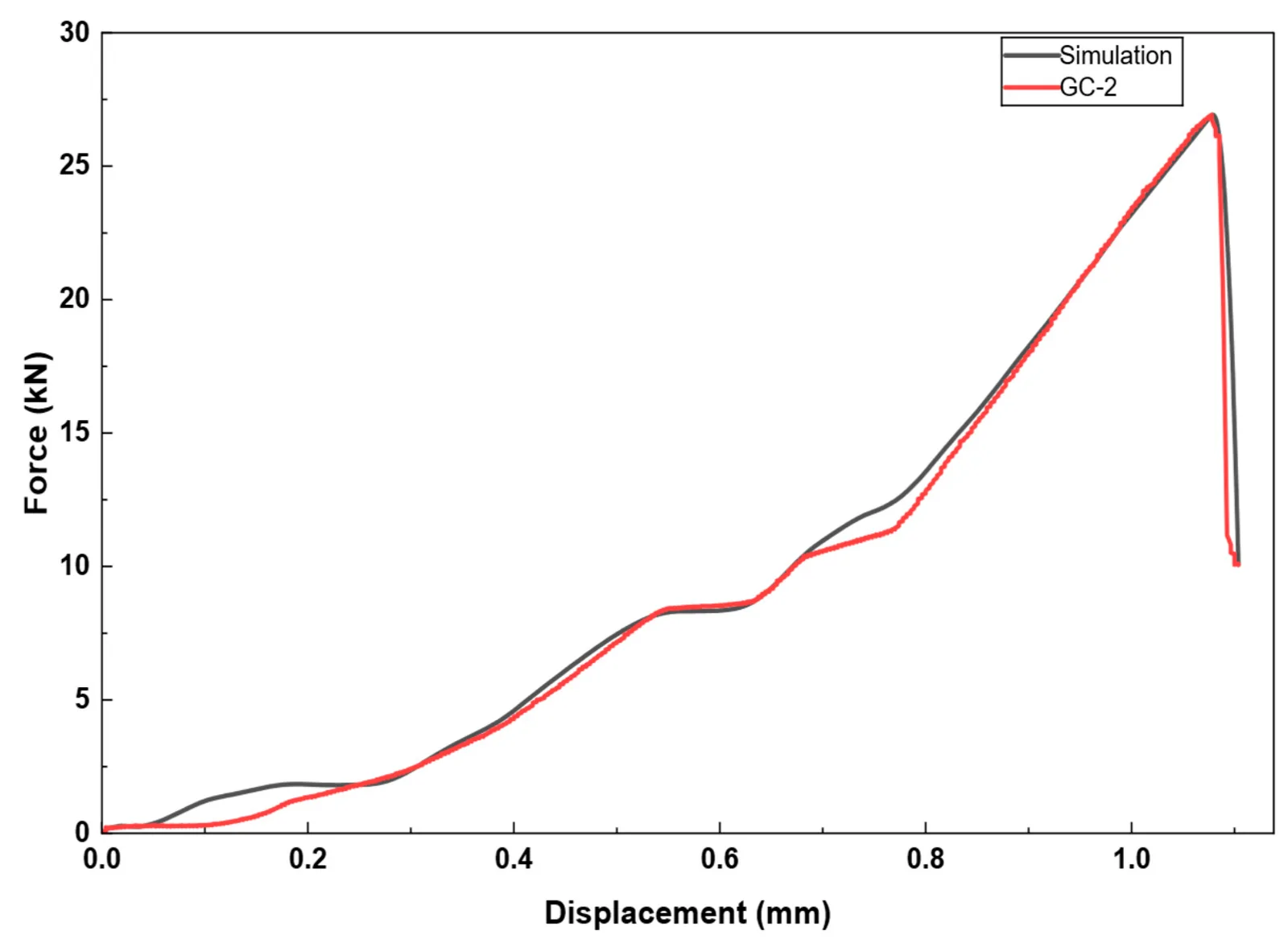
Experimental and numerical CAI load–displacement comparison for 30° QSI tested specimen.

Figure 19 and Figure 20 compare the experimental and simulated CAI load–displacement curves. The numerical model for the 15° QSI tested specimen predicted a peak CAI load of 19.43 kN, which corresponds to a residual compressive strength of 60.72 MPa. This value is very close to the experimental average, indicating that the model captured the compressive response of the 15° QSI tested laminate successfully. The simulation for the 30° QSI tested specimen predicted the peak CAI load of 26.93 kN and residual compressive strength of 84.16 MPa. This is close to the experimental average value of 26.30 kN and 82.18 MPa, indicating good agreement between simulation and experiment. The good agreement between experimental and numerical peak loads showed that the finite element model was able to predict the residual compressive capacity of the indented GC laminates with reasonable accuracy. The model also identified the primary failure locations and damage concentration around the indentation zone. Nevertheless, differences in the shape of the curve and displacement response may occur due to simplifications of the material properties, damage evolution laws, contact behavior, and boundary conditions.

The overall simulation results are consistent with the experimental ones and validate the effect of the indentation angle on the CAI behavior of the GC hybrid woven laminate. The model predicted a higher residual strength for the 30° case, consistent with the experimental trend. This suggests that the proposed numerical approach can be adopted to study the relationship between indentation-induced damage and residual compressive performance.

Table 5 summarizes the test and simulated results for comparisons. It is shown that the compression performance of the 30° QSI tested specimens was better than that of the 15° QSI tested specimens after indentation damage. The average residual compressive strength increased from 60.72 MPa to 82.18 MPa when the indentation angles increased from 15° to 30°, an improvement of about 35.36%.

The images taken after failure confirmed that the failure started in the area of the indentation damage and propagated by matrix cracking, fiber fracture, and transverse crack growth. The 15° QSI tested specimens exhibited more localized damage and lower residual strength, while the 30° QSI tested specimens exhibited more distributed damage and higher residual compressive capacity.

The numerical simulations gave good agreement with the experimental CAI results. The predicted peak loads and residual strengths were in good agreement with the experimental values, and the simulated damage contours confirmed that the indentation region controlled the compressive failure process. Thus, the combined experimental and numerical results show that the indentation angle has a significant influence on the QSI energy absorption, damage morphology, and residual compressive performance of the GC hybrid woven composite laminates.

## 4. Conclusions

The present work investigates the effect of oblique QSI angle on damage mechanisms and CAI performance of GC hybrid woven composite laminates. Hemispherical indentation tests were performed at 15° and 30° and were followed by damage characterization, CAI testing, and finite element simulation. The finite element model was validated primarily using force–displacement response and qualitative agreement with the experimentally observed damage locations. CT measurements were also used as representative supporting evidence for damage morphology. The following conclusions may be drawn based on the reported experimental and numerical results:

(1) The GC laminates showed similar peak indentation resistance at the two oblique indentation angles. The average peak load of QSI was 10.57 kN for 15° indentation and 10.66 kN for 30° indentation. This indicated that increasing the indentation angle from 15° to 30° had little effect on the maximum indentation load. Indentation angle had a greater effect on deformation and energy absorption than the peak load. The displacement at peak load increased from 6.87 mm at 15° indentation to 12.92 mm at 30° indentation. Likewise, the total absorbed energy rose from 70.86 J to 102.44 J. This suggests that the 30° indentation condition allowed for higher deformation and progressive energy dissipation before failure.
(2) Observation of surface damage and CT results showed that the damage morphology was dependent on the indentation angle. The specimens at 15° indentation exhibited more compact localized damage around the indentation region, while the specimens at 30° indentation showed a wider, more directional damage pattern. The observed behavior was assigned to the increased tangential force component introduced at the higher oblique angle. Representative CT measurements showed that the b-30-3 specimen had a larger damage diameter and a slightly higher damage depth than the b-15-3 specimen. However, since CT inspection was conducted only for selected specimens, these results were taken as supporting damage evidence, not as statistical averages.
(3) The CAI results showed that the residual compressive performance of 30° QSI tested specimens was better than that of 15° QSI tested specimens. The average residual CAI strength increased from 60.72 MPa to 82.18 MPa when the indentation angle increased from 15° to 30°, an increase of 35.36%. This indicated that although the 30° indentation resulted in increased deformation and energy absorption during QSI, the damage created was not as severe as the more localized damage created at 15° indentation, which was more critical to the collapse of the compression. Post-CAI failure revealed that the failure was initiated near the indentation-damaged region and propagated by matrix cracking, fiber fracture, and delamination growth. The localized indentation damage in the 15° QSI tested specimens seemed to cause a greater stress concentration during compression, whereas the more distributed damage in the 30° QSI tested specimens permitted a higher residual load-bearing capacity.
(4) The numerical simulations reproduced the main experimental trends, including the QSI force–displacement response, localized stress concentration, damage initiation near the indentation region, and the higher CAI strength of the 30° case. The numerical model could predict the experimental CAI peak loads with good accuracy. Further calibration of the contact friction, cohesive properties, and damage degradation parameters is recommended to enhance the prediction accuracy, particularly for higher oblique indentation angles.

It is found that within the tested oblique angle range, increasing the indentation angle from 15° to 30° influenced the deformation response, energy absorption, and damage morphology of the laminates. For the GC laminate studied in this research, increasing the indentation angle from 15° to 30° did not significantly change the indentation peak load but increased the deformation capacity, energy absorption, and CAI residual strength. The reported results provide useful insights for the damage-tolerant design and numerical evaluation of hybrid woven composite structures under oblique contact loading. Another limitation of the present study is that indentation was performed on initially unloaded specimens. In practical aerospace structures, localized contact events may occur while components are subjected to in-service tensile or compressive loads. Future work will investigate the interaction between external preloading and oblique indentation to better represent operational conditions.

## Figures and Tables

**Table 1:** Mechanical properties of the composite materials.

Property	Glass Woven Laminate	Carbon Woven Laminate
Longitudinal modulus (*E*_1_, *E*_2_)	19.03 GPa	57.74 GPa
Out-of-plane modulus (*E*_3_)	5.63 GPa	7.7 GPa
In-plane Poisson’s ratio (*v*_12_)	0.11	0.07
Out-of-plane Poisson’s ratios (*v*_13_, *v*_23_)	0.32	0.27
In-plane shear modulus (*G*_12_)	3.42 GPa	9.66 GPa
Out-of-plane shear moduli (*G*_13_, *G*_23_)	2.00 GPa	6.40 GPa
In-plane shear strength (*S*_12_)	69.76 MPa	141.72 MPa
Out-of-plane shear strengths (*S*_13_, *S*_23_)	15.53 MPa	70 MPa
Tensile strength, in-plane (*X*_T_, *Y*_T_)	188.72 MPa	609.67 MPa
Compressive strength, in-plane (*X*_C_, *Y*_C_)	74.85 MPa	533.59 MPa
Tensile strength, out-of-plane (*Z*_T_)	15.15 MPa	50 MPa
Compressive strength, out-of-plane (*Z*_C_)	42.6 MPa	150 MPa

**Table 2:** Summary of QSI test results for GC laminates.

Specimen	Peak Load (kN)	Displacement at Peak Load (mm)	Total Energy Absorption (J)	Energy Absorbed Till Peak Load (J)
GC-15–1	10.29	7.45	66.83	36.67
GC-15–2	11.80	7.30	75.18	42.78
GC-15–3	9.63	5.87	70.57	29.96
GC-15° Avg ± SD	10.57 ± 1.13	6.87 ± 0.89	70.86 ± 4.18	36.47 ± 6.43
GC-30–1	10.28	11.53	96.98	52.36
GC-30–2	10.20	12.58	108.07	62.49
GC-30–3	11.50	14.64	102.28	74.64
GC-30° Avg ± SD	10.66 ± 0.73	12.92 ± 1.57	102.44 ± 5.55	63.16 ± 11.15

**Table 3:** CT-based damage measurements for selected hemispherical indentation specimens.

Specimen	Defect Ratio (%)	Damage Volume (mm^3^)	Damage Surface Area (mm^2^)	Damage Diameter (mm)	Damage Depth (mm)
b-15-3/GC-15°	6.872	301.526	564.164	16.791	4.359
b-30-3/GC-30°	5.031	315.217	561.433	20.366	4.594

**Table 4:** Summary of CAI experimental results for GC laminates.

Specimen	Peak CAI Load (kN)	Displacement at Peak Load (mm)	Residual CAI Strength (MPa)
GC-15–1	20.87	0.789	65.22
GC-15–2	17.99	0.670	56.22
GC-15° Avg ± SD	19.43 ± 2.04	0.730 ± 0.084	60.72 ± 6.36
GC-30–1	25.67	0.770	80.22
GC-30–2	26.92	1.078	84.13
GC-30° Avg ± SD	26.30 ± 0.88	0.924 ± 0.218	82.18 ± 2.77

**Table 5:** Comparisons between experimental and simulated CAI results.

Case	Peak CAI Load (kN)	Residual CAI Strength (MPa)	Difference from Experimental Results
GC-15° Experimental (Avg)	19.43	60.72	—
GC-15° Simulation	19.43	60.72	0.00%
GC-30° Experimental (Avg)	26.30	82.18	—
GC-30° Simulation	26.93	84.16	+2.40%

## Data Availability

The original contributions presented in this study are included in the article. Further inquiries can be directed to the corresponding authors.
